# Perspectives on managing innovation readiness in long-term care: a Q-methodology study

**DOI:** 10.1186/s12877-024-05572-3

**Published:** 2024-12-19

**Authors:** Monique W. van den Hoed, Ramon Daniëls, Audrey Beaulen, Jan P. H. Hamers, Job van Exel, Ramona Backhaus

**Affiliations:** 1 Department of Health Services Research, Faculty of Health, Medicine and Life Sciences, CAPHRI Care and Public Health Research Institute, Maastricht University, Duboisdomein 30, Maastricht, 6229 GT The Netherlands; 2 Living Lab in Ageing and Long-Term Care, Duboisdomein 30, Maastricht, 6229 GT The Netherlands; 3 Zuyd Expertise Centre for Innovative Care and Technology Research Centre for Assistive Technology in Health Care, Zuyd University of Applied Sciences, Nieuw Eyckholt 300, Heerlen, 6419 DJ The Netherlands; 4 Erasmus School of Health Policy & Management (ESHPM), Erasmus University Rotterdam, Burgemeester Oudlaan 50, Rotterdam, 3062 PA the Netherlands; 5 Erasmus Centre for Health Economics Rotterdam (EsCHER), Erasmus University Rotterdam, Burgemeester Oudlaan 50, Rotterdam, 3062 PA the Netherlands; 6 Fliedner Fachhochschule Düsseldorf, Geschwister-Aufricht-Straße 9, 40489 Düsseldorf, Germany

**Keywords:** Innovation readiness, Q-methodology, Long-term care, Organizations, Management.

## Abstract

**Background:**

The scarcity of resources in long-term care demands more than ever that organizations in this sector are prepared for innovation to ensure affordable access to care for older adults. Organizations that are innovation ready are more capable of implementing innovations. Therefore, a better understanding of how stakeholders view innovation readiness in long-term care can provide actionable strategies to enhance their innovative capacities. ‘Innovation readiness’ indicates the level of maturity of an organization to succeed in any type of innovation. Our study explored perspectives among stakeholders on what they consider important for organizations in long-term care for older adults to be innovation ready.

**Methods:**

Q-methodology, a mixed-methods approach, was used to investigate the perspectives of 30 stakeholders connected to long-term care for older adults in the Netherlands: academics, (top)management, innovation managers, client representatives, staff, and consultants. Stakeholders were asked to rank 36 statements on innovation readiness on importance. Statements were extracted from literature research and qualitative interviews. Thereafter in the post-interviews stakeholders explained their ranking and reflected on the statements. By-person factor analysis was used to identify clusters in the ranking data. Together with the qualitative data from follow-up interviews, these clusters were interpreted and described as perspectives of the stakeholders.

**Results:**

Four distinct perspectives were identified on what they consider important for innovation readiness in long-term care: (1) ‘supportive role of management’ (2) ‘participation of the client (system) and employees’ (3) ‘setting the course and creating conditions’ and (4) ‘structuring decision-making, roles and responsibilities’. The 36 statements represented a complete overview of innovation readiness factors. No additional innovation factors to those previously identified in the literature emerged from the interviews.

**Conclusions:**

Stakeholders agree that all factors contributing to innovation readiness of long-term care organizations for older adults are accounted for. The variety of perspectives on what is most important shows there is no agreement among stakeholders about a fixed route toward innovation readiness. However, stakeholders suggested a temporal order of the innovation readiness factors, preferably starting with formulating the innovation ambition. This study’s results could contribute to developing an assessment tool to deliver a structured approach for managers to assess the innovation readiness of their organization.

**Registration:**

The study received ethical approval on April 13, 2022 from the Medical Ethics Board of Zuyderland Medical Center in the Netherlands with the number METCZ20220036.

**Supplementary Information:**

The online version contains supplementary material available at 10.1186/s12877-024-05572-3.

## Background

Long-term care organizations for older adults (e.g. care homes, nursing homes, assisted living facilities, residential aged care facilities) provide a range of services, including medical, transitional, and nursing care, housing, personal care, assistance, and social services to older adults who cannot live independently [1]. Dutch long-term care is largely funded through mandatory public health insurance and is increasingly focusing on person-centered care, integrating technology (e.g., eHealth, telecare), and promoting self-management to enhance the quality of life [2]. Complex care demands, nursing staff shortages, and scarcity of resources [3] demand more than ever that long-term care organizations are prepared for innovation to ensure affordable access to care for older adults [4–8]. Greenhalgh et al. [9] see innovation as “a novel set of behaviors, routines, and ways of working that are discontinuous with previous practice, are directed at improving health outcomes, administrative efficiency, cost-effectiveness, or user experience and that are implemented by planned and coordinated actions.” Organizations that are innovation ready are more capable of implementing innovations [9–11]. Thus, ‘innovation readiness’ indicates the level of maturity of an organization to succeed in any type of innovation [12]. Innovation strategies such as utilizing technologies and implementing integrated care models are aimed at improving the care quality and efficiency of their services. Considering the challenges they face, long-term care organizations for older adults might benefit from more knowledge about how to become innovation ready [13–15].

The significance of promoting innovation within long-term care organizations has gained widespread acknowledgment both in the literature and in day-to-day practice [9, 16]. Recent literature addresses innovation readiness within healthcare with a variety of words such as ‘capacity for innovation’ [17–20], ‘innovation capacity’ [21], ‘capacity to innovate’ [22–24], ‘ability to innovate’ [25–27], ‘organizational innovativeness’ [22], ‘organization’s innovation ability’ [28], ‘innovation performance’ [29, 30], ‘innovativeness of organizations’ [31], ‘organizational innovation’ [32] and ‘organization’s innovative potential’ [33]. Insight into the conditions on how to become innovation ready remains relatively scarce [31, 34, 35]. A recent study proposed a framework comprising five main factors enabling innovation readiness of long-term care organizations for older adults: 1) strategic course for innovation 2) innovation journey 3) leadership for innovation 4) learning for innovation and 5) innovative organizational culture [36]. However, the importance of these factors seems to vary within the long-term care sector [31, 34, 36] and, therefore, it is desirable to gain a deeper understanding of the perspectives of stakeholders in this sector.

According to Nolte [13], innovating in long-term care takes place on a multi-level organizational perspective, requiring collaboration between locations, disciplines, teams, and employees, who all may have different challenges and distinct perspectives on how to become innovation ready [37]. Research exploring the perspectives of the various stakeholders in long-term care on innovation readiness has not yet been undertaken [38, 39]. Thus, our current study aimed to address this gap by investigating the perspectives of stakeholders with a role in innovating in long-term care organizations on what is important for organizations in this sector to be innovation ready. In addition, we tested the comprehensiveness of the list of innovation readiness factors previously identified in the literature [36]. The study had two research questions: 1. What are the prevailing perspectives on factors enabling innovation readiness among stakeholders with a role in long-term care for older adults? 2. Are there additional factors that contribute to innovation readiness?

## Methods

### Design: Q-methodology

We used Q-methodology to identify and describe perspectives on what is important for innovation readiness among stakeholders in long-term care for older adults. Q-methodology is a mixed-methods approach for systematically studying perspectives, opinions and beliefs. The ‘Q’ stands for ‘quantification’ of subjective data, with which the perspectives of individuals can be analyzed and interpreted in a systematic and structured manner [40–42]. Q-methodology involves a card sorting activity to rank a set of statements (on innovation readiness), which are analyzed using by-person factor analysis to identify shared viewpoints in the data [41]. Q-methodology is increasingly used in healthcare research and other disciplines for identifying and comparing individuals’ and groups’ perspectives [41, 43, 44]. Our study was conducted in four main steps, as common to Q-methodology studies: (1) development of the statement set; (2) selection of respondents; (3) card-sorting and post-interview; (4) analyses and interpretation.

1. **Development of the statement set**

**Fig. 1:**
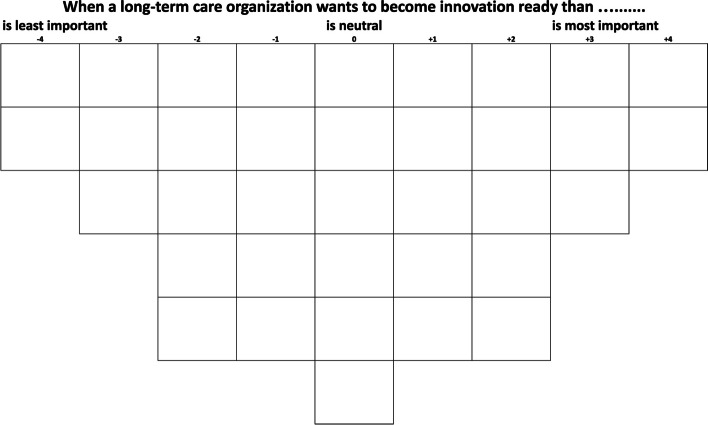
**Sorting grid**

To capture the full range of perspectives on a specific topic adequately, the statement set presented to respondents should have good coverage of the subject of interest [40]. For the development of a comprehensive overview of factors potentially contributing to the innovation readiness of organizations in long-term care, we used the results of a recent scoping review [12] and an interview study [36] on this topic, the scientific literature discussed here above, and statements from opinion leaders in the Netherlands related to innovation readiness [45, 46]. Altogether, this resulted in a first set of 112 possible statements (the concourse [47]). To make sure all the potentially important factors for innovation readiness were covered, these statements were categorized according to the five main factors from the innovation readiness framework of Van den Hoed et al. [36]. Via a group session with healthcare researchers of the Living Lab in Ageing and Long-Term Care at Maastricht University in the Netherlands and iterative discussions within the research team, a pilot set of 36 statements was selected for the study (the Q-set [47]). Face-to-face pilot interviews were organized to test the interview materials, including the statements (printed on cards), the sorting grid (Fig. 1), the step-by-step instructions for conducting the card sorting exercise and the interview guide, with five respondents (i.e., one top manager, one client representative, three innovation managers; duration approx. 60 min). The aim of the pilot was to evaluate if the statement set was comprehensive [48], whether all the interview materials were clear and accurate, and the time taken to complete the Q-sort interview. This pilot resulted in the rewording of four statements. The final set of 36 statements covering potentially important factors for innovation readiness of organizations in long-term care for older adults is presented in Table 1.

2. **Selection of respondents**

We anticipated that how stakeholders perceive the relative importance of factors contributing to innovation readiness might vary by function, role, geographical location, low to high level of experience with innovating [49], and size of the organization [50]. Consequently, respondent recruitment aimed for diversity on these characteristics by approaching the following stakeholder groups: (1) academics, (2) (top) management, (3) innovation managers, (4) staff, (5) client representatives, and (6) consultants guiding organizations in making them ‘innovation ready’. Further, we sought access to respondents in smaller and larger organizations spread over the Netherlands. The sex and gender of the respondents were not taken into account in the design of the study, as no potential implications of sex and gender on the study results and analyses were expected [51]. Potential respondents were identified through a purposive sampling [52] approach. They were initially recruited through the networks of the research team and, next, using snowball sampling via these initial contacts. Identified potential respondents were invited by personal contact, email (if an email address was publicly available), or social media (LinkedIn). All contacted respondents were asked if they were familiar with innovation readiness, the topic of this study. We included respondents if they (1) were researchers or professionals with academic or practical knowledge in the field of innovation in long-term care for older adults and (2) were able to articulate perspectives on factors important for innovation readiness of long-term care organizations for older adults in the Netherlands and (3) had a professional role in innovating in long-term care organizations for older adults.

3. **Card-sorting and post-interview**

Respondents conducted the card-sorting task online via Qmethod software, a computerized web-based application customized with content by the interviewer, allowing respondents to sort the statements online [53]. Conducting Q-methodology studies online is feasible, especially due to increasing experience with online applications and software developments since the COVID-19 pandemic [54, ]. Research questions, instructions, statement cards and sorting grid were presented via the software in real-time to respondents. The respondent was asked to share the screen with the interviewer during the card-sorting task and the interviewer gave verbal instructions for the task and answered questions, if needed. After the respondents were provided with the research question they were asked to rank the statements. The sorting grid (Fig. 1) consisted of 36 items with a numerical ranking from least important (− 4) to most important (+ 4) in a nine-point distribution as is suggested for statement sets of 40 items or less to facilitate the ranking [40]. The instruction for the task was presented on top of the sorting grid. Respondents were encouraged to also comment (to think-aloud) while they were sorting the cards to provide valuable information for the interpretation of the results [55]. After the respondent was finished with the sorting, the results were captured via a screenshot and in the Qmethod software. The post-sorting interviews, conducted online via Zoom (one-to-one) (see Additional files 1–6), covered two topics: (1) the reasoning behind the placement of cards, including the extremes of the sorting grid (scoring − 4, − 3, + 3, and + 4) and (2) innovation readiness factors that respondents thought were not included the statement set. The post-sorting interviews were conducted as an approach to access more in-depth information [56] and encourage the respondent to tell ‘the story’ in their own words [57, 58]. The post-interviews (with the consent of each respondent), were recorded and transcribed.

4. **Analyses and interpretation**

The quantitative part of the analyses consisted of a by-person factor analysis using common techniques in Q-methodology (i.e., centroid extraction, varimax rotation [59]) and was performed to identify groups of respondents who had ranked the statements in a similar way. The assumption made is that if respondents have a similar opinion, they will rank the set of statements in a similar way. Consequently, these factors can be interpreted as shared perspectives on what is important for organizations in this sector to be innovation ready. The number of factors to retain for interpretation was selected on the basis of factors having an Eigenvalue larger than one, a minimum of two respondents loading statistically significant (*p* < .05), the cut-off was 0.33 : 1.96*(1÷√No. of items in q-set)=1.96*(1 ÷ 6)=0.33) and a coherent interpretation [40]. For each of the identified factors, an idealized ranking of the statements was calculated (factor arrays [47]), which represents how a respondent perfectly correlated with this perspective, would have ranked the statements (see Additional files 7–10). This involved calculating a weighted average ranking of the statements for respondents who are statistically significantly linked to this particular factor. The composite rankings of the statements of the four factors (Table 1) complemented with the qualitative data (consisting of the explanations of the respondents, statistically significantly associated with that perspective, given during the post-interview) were used to interpret and describe the factors as perspectives on factors enabling innovation readiness [60]. The first interpretation of the perspectives was based on the characterizing, distinguishing and consensus statements for each perspective. Characterizing statements are those ranked by the respondents as most important (with a + 4, or + 3 score) or as least important (with a −4 or −3 score) in the composite ranking. Distinguishing statements are those that were ranked statistically significantly in a factor as compared to the other factors (denoted with a star* in Table 1). Consensus statements are those that are ranked similarly across factors (denoted with a plus + in Table 1).

This first interpretation was then further refined using the explanations provided by respondents associated with the factor, and citations from these qualitative materials were added to the description of the perspectives for illustration purposes. The data were analyzed using KADE [61].

### Quality assurance

The study received ethical approval from the Medical Ethics Board of Zuyderland Medical Center in the Netherlands with the number METCZ20220036. Permission to conduct the interviews for this study was granted by each respondent personally. Respondents were made aware of the study objectives, and written informed consent was obtained from respondents before the beginning of the interviews. A process logbook was kept by the first author to ensure that essential decisions were registered in a retrievable way from the start until the end of the research. This enabled the research team to monitor the progress and decision-making processes during the study. The logbook is stored on the UMserver, with access for the study team, and includes descriptions of important moments, decisions and solutions/actions undertaken.

The final data (on which the reported analyses are based) is stored on the UMserver and can be made available on request. In the Q-method software, the researcher does not have access to the respondent’s Personal Identifiable Information (PID). The software does not record the IP addresses of respondents.

## Results: interpretation of perspectives

In total, 30 stakeholders connected to long-term care participated in this study between April and June 2023 (Table 2). Respondents expressed that the set of 36 statements (Table 1) represented a complete overview of factors contributing to innovation readiness of long-term care organizations for older adults. The by-person factor analysis resulted in four factors, with 8, 5, 5 and 8 of the 30 respondents statistically significantly associated with them, respectively. Together the factors explained 46% of the variance in the ranking data (factor 1 to 4 respectively explained 22%, 9%, 9%, and 6% of the variance) and correlations between factors varied between 0.01 (factor 2 vs. factor 3) and 0.47 (factor 1 vs. factor 4). The first factor, which showed correlations between 0.19 and 0.47 with the other three factors will be presented first. Factors 1 vs. factor 3 and factor 1 vs. factor 4 were statistically significant respectively 0.36 and 0.47. Three respondents associated with more than one perspective and one respondent with a negative loading on factor 2 were not included in the computation of the composite sorts. After inspecting the composite sorts and the qualitative data from the interviews, the research team concluded that each one of the four factors represented an interesting and distinct perspective on innovation readiness. The composite rankings of the statements for these four perspectives are presented in Table 1 and shown in Additional files 7-10. The consensus statements did not highlight specific differences or similarities between the perspectives.

Below, we describe each perspective. Quotes from respondents are added in italics, followed by the number of the respondent. The first number in the brackets refers to the number of the statement from Table 1 and the second number refers to numerical ranking given to the statements in the sorting grid (Fig. 1) consisting of least important (− 4) to most important (+ 4).

**Table 1:** Statements and ranking scores on the relative importance of factors contributing to innovation readiness [36]

Statement set		Perspectives			
		1. Supportive role of management	2. Participation of the client (system) and employees	3. Setting the course and creating conditions	4. Structuring decision-making, roles and responsibilities
**1. Strategic Course for Innovation**					
1	Formulate an innovation ambition	1*	−3*	4	4
2	Determine innovation theme(s)	0	0	2*	4*
3	Describe the organization’s definition of innovation	−3	−4	1	−1
4	Allocate budget for innovation	2	1	3*	2
5	Create a multi-annual plan for innovation	−1	−4*	0	−2*
6	Make agreements about position and tasks of employees engaged in innovation	−2*	0*	−4*	3*
7	Set up innovation team(s) / unit(s)	0	−3	0	−2
8	Prepare technical infrastructure for innovation	0	−2	−3	1
9	Make innovation knowledge (gained in projects) available	−1	0	−2*	−4*
10	Make a communication plan for innovation	0	−1	1	−1
**2. Innovation Journey**					
11	Define the decision-making steps in the innovation process	−1	1	0	2*
12	Make a toolbox with innovation instruments available	−3	−3	−2	0*
13	Organize an innovation process (from idea to implementation)	−2	0	2	2
14	Have an overview and insight into the progress of innovations	1	−1	1	0
15	Involve family and relatives while innovating	−4*	4*	1	1
16	Actively involve health care professionals in the innovation process	4	4	2	2
17	Exchange innovation knowledge with healthcare and knowledge institutions +	0	0	−2	−1
18	Monitor national innovation developments and trends	−2	−1	1*	−3
19	Collaborate with external partners on innovation themes	1	1	2*	−2*
20	Formulate a vision on learning from and about innovation	1*	−1*	3*	−2*
21	Organize education aimed at learning how to innovate	−3	2*	−2	−2
22	Compose innovation team(s) interdisciplinary	2	−2	2	−1*
23	Support middle management with knowledge for their role in innovating	3*	0	−4*	1
**3. Leadership for Innovation**					
24	Clear role for middle management in the field of innovating	2	−1*	−3*	3
25	Appreciate employees for their commitment to innovation	2	3	−1*	2
26	Middle management creates an attractive innovation climate for employees	3*	1	−1	0
27	Board communicates that innovation is an organization priority	4	−2*	4	3
28	Clear role for employees in the field of innovating	2	2	−2*	0
**4. Learning for Innovation**					
29	Reflect on innovation readiness of the organization +	−2	−2	−1	−1
30	Capture and evaluate learning experiences around innovation	−2	1*	−1	−3*
31	Set up physical spaces in the organization for innovation activities	−4	−2*	−3*	−4
32	Encourage employees to start with innovating themselves	−1	2*	0	0
**5. Innovative Organizational Culture**					
33	Have the courage to experiment	3	3	3	1*
34	Learn from failure and mistakes	0	2*	−1	−3*
35	Take time to learn	−1	3*	0	1
36	Learn from each other in the field of innovating +	1	2	0	0

*Distinguishing statement (*p* < .05) for that perspective - are those statements that are found to be statistically significantly different from other statements within the other perspectives when it comes to how they are ranked by the respondents

+ consensus statement - are those statements that do not distinguish between the various perpectives

Scores range between − 4 and + 4 correspond to the columns of the sorting grid (see Fig. 1): −4 concerns ‘least important’; 4 concerns ‘most important’

**Table 2:** Characteristics of participating respondents

No.	Respondent group	Type of Organization	Size of organiza-tion^d^	Main role of respondent
1	client representatives	Long-term care^a^	medium^b^	Client representative
2	innovation managers	Long-term care^a^	medium^b^	Project leader innovation
3	innovation managers	Innovation program long-term care	(n.a.)	Regional program manager innovation
4	client representatives	Long-term care^a^	medium^b^	Chair of the central client council
5	consultants	Consultancy		Consultant long-term care
6	academics	Expertise Centre long-term care		Senior advisor innovation
7	consultants	Independent		Innovation expert healthcare
8	(top)management	Innovation Fund for Long-term care	small^c^	CEO
9	(top)management	Long-term carea	medium^b^	Director HRM & Innovation
10	staff	Long-term care^a^	small^b^	Program manager processes & innovation
11	staff	Long-term care^a^	medium^b^	Program manager innovation & development
12	academics	University		Professor management & organization long-term care
13	client representatives	Client support organization		Director/coordinator
14	staff	Long-term care^a^	medium^b^	Policy advisor research & development & innovation
15	innovation managers	Hospitals, home care, long-term care^a^	large^b^	Manager innovation & information & automation
16	academics	University		Program Director Executive Master of Health
17	consultants	Independent		Consultant
18	academics	University of applied sciences		Lector in Long-term care
19	(top)management	Long-term care^a^	large^b^	CEO
20	(top)management	Long-term care^a^	medium^b^	CEO
21	consultants	Consultancy	medium^b^	Advisor
22	(top)management	Long-term care^a^	small^b^	Chairman of the Board of Directors
23	innovation managers	Long-term care^a^	large^b^	Program manager innovation and e-health
24	staff	Hospitals, home & long-term care^a^	large^b^	Head of Scientific Research
25	consultants	Independent		Consultant
26	staff	Long-term care^a^	medium^b^	Manager information management
27	academics	University of applied sciences		Professor
28	client representatives	Long-term care^a^	medium^b^	Chair of the central client council
29	innovation managers	Association for long-term care^a^		Senior advisor digitizing & innovation
30	client representatives	Long-term care^a^	small^b^	Coordinator client councils and network of volunteers

^a^Long-term care organization for older adults providing medical, transitional and nursing care, housing, personal care, assistance, and social services to older adults who cannot live independently

^b^Annual reports 2022

^c^Website of organization

^d^Turnover small €0–100 M, medium €100–200 M, large €200 M and more

### Perspective 1: supportive role of management

Central in this perspective is the opinion that the top of the organization has to articulate the innovation strategy (#27,+4) (#1, + 1*) and management has to simultaneously facilitate the climate to become innovation ready (#26, =+3*). *‘Formulating a vision on innovation starts at the top and management must create conditions for execution’* (respondent 10, staff member). In this perspective, (top)management roles are outlined by the respondents as ‘defining the innovation course’, ‘creating the climate and conditions’ and ‘communicating about innovation.’ *‘(Top)management must point the organization’s compass into the right direction’* (respondent 19, (top)managment); *‘People only participate if the board clearly communicates about the innovation purpose via annual meetings and intranet’* (respondent 24, staff member).

A significant aspect is that respondents of this perspective consider the active involvement of healthcare professionals and staff important in all steps of the innovation process (#16,+4) as they know whether or not an innovation will work; *‘The board sets the innovation course involving employees in that process because they determine whether innovation can be promising or successful’* (respondent 5, consultant). Therefore, respondents express that roles and tasks for employees engaged in innovation should be clear (#28, + 2), but not fixed (#6, −2*).

The role of middle management is to create an attractive innovation climate by building trust, creating a safe environment, giving backup, communicating and prioritizing team activities (#26, + 3*). *‘Managers are needed to translate and communicate the innovation compass (determined by the board) into a roadmap for their team.’* Besides the importance of strategy and planning, respondents emphasized to make room for trial and error. Which requires management to have courage (#33,+3*). *‘Also part of innovation is having the courage to experiment and allow yourself some failures’* (respondent 19, (top)managment).

Respondents express that learning from and about innovation is important. Besides attention for a vision on learning how to innovate (#20, + 1*) they express that middle management should be supported with knowledge for their role in innovating (#23,+3*). *‘Support management to help them to overcome problems and have conversations with their team to find out: what do they run into?’* (respondent 19, (top)managment). Management education should support them to facilitate an attractive innovation climate (#26,=3*).*‘Managers need to encourage healthcare professionals to come up with innovative ideas. They need the knowledge to facilitate this process of innovation and learning’* (respondent 11, staff member). Providing a toolbox of innovation tools (#12,−3) and learning how to innovate (#21, −3) are not seen as a meaningful approach. *‘First: get employees on board*,* the toolbox will come at a later stage’* (respondent 9, (top)managment).

Of all the perspectives expressed, this one expresses a deliberate trade-off on the involvement of family and relatives while innovating (#15, −4*) between relevance and doing it ‘by default’. They mention that family and relatives should only be involved when it is seen as relevant to their situation. *‘Involving family depends on the type of innovation. Depending on the expected impact*,* you will either inform or involve them’* (respondent 5, consultant). Setting up physical spaces in the organization for innovation activities (#31, −4) is not seen as an added value by the respondents. They firmly believe that if organizations want to innovate, support needs to be organized close to the workplace. *‘Physical spaces*,* you don’t have to have them*,* as most innovations are integrated into current work processes’* (respondent 9, (top)managment).

### Perspective 2: participation of the client (system) and employees

Central in this perspective is the opinion that active participation of both the client (system) and employees in the innovation activities of the organization (#15,+4*) (#16, + 4) are most important. Respondents indicate that innovation should be aimed at the quality of care for the person who needs care, and therefore family and loved ones have to be involved in innovation (#15,+4*). *‘The essence is that organizations innovate to make it better for people receiving care*,* for people providing care*,* and for the network around it’* (respondent 4, client representative). Respondents most strongly agree (compared to perspectives 1, 3 and 4) that innovation should foremost be bottom-up based on the ideas and needs of the employees and less top-down (#1, −3*) (#27, −2*). Respondents strongly value, in line with perspective 1, the active involvement of healthcare professionals in the innovation process (#16, + 4) as *‘The willingness to innovate of healthcare professionals therein lies the essence of innovation readiness’* (respondent 1, client representative).

Similar to perspectives 1 and 3, respondents with perspective 2 believe that an organization has to appreciate employees for their commitment to innovation (#25, + 3) as they are the ones who make it happen. *‘The commitment and attitude of the organization are important and determine whether an innovation can be successful’* (respondent 21, consultant). Therefore, the organization should reward employees for their efforts and for the struggle that comes with innovation (#33, + 3). *‘If employees feel that they are allowed to innovate and experiment*,* they will want to keep doing it. If they are punished or reprimanded for not moving fast enough*,* a negative mode on innovating can arise’* (respondent 21, consultant).

Respondents are not in favor of setting up innovation teams (#7,−3), as innovation has to be done with all those who will use and work with it. *‘It is best to involve everyone who wants and can and as much and as early as possible in innovations. Then you immediately know whether it works or not’ (*respondent 21, consultant) In line with this, respondents holding this perspective strongly oppose managerial actions such as making an innovation definition (#3,−4) and creating a multi-annual plan for innovation (#5,−4*) as it does not help the employee in the workplace. *‘Innovation is dynamic*,* which is opposite to a plan in which you commit to what you have planned out. You can commit to doing it*,* but not on the how and the when’* (respondent 13, client representative).

Respondents ranked statements related to stimulating and learning about innovation for employees as significantly more important than in perspectives 1,2, and 4 (#35,+3*) (#32,+2*) (#34,+2*) (#21,+2*). They consider taking time as an organization to learn how to innovate most important (#35, + 3*) because innovating concerns behavior change and making new routines costs time. Therefore, they consider it essential to have realistic expectations and to not expect results from the innovation processes too quickly (#35, + 3*). *‘Healthcare professionals and staff should be given space to experiment*,* make mistakes*,* and take their time as innovating never happens overnight’* (respondent 21, consultant). Furthermore, respondents express that encouraging employees to start with innovation themselves (#32,+2*) and learning from mistakes (#34,+2*) adds to a favorable innovation culture. *‘At the organizational level*,* you can facilitate anything*,* but you also have to encourage people to feel free to just do it. That way they feel that they are in the lead to improve their work’* (respondent 30, client representative). Respondents indicate that education focused on learning to innovate (#21,+2*) should be determined by the individuals and teams based on what they need in their work and context and not predetermined by the organization (#20,−1*).

### Perspective 3: setting the course and creating conditions

Perspective 3 focuses on organizational factors that are either supportive or conditional in becoming innovation ready. Respondents indicate the importance of preparing the organization’s innovation direction (#1,+4) (#2,+2*) and organizing it to enable the envisioned direction. *’Formulating an innovation ambition ensures a clear perspective that can be shared in the organization’* (respondent 12, academic). Furthermore, aspects of organizing innovation deployment, to enable the strategic innovation course, are ranked in this perspective as significantly more important than in the other three perspectives. Respondents indicate that a vision of learning from and about innovation and a program to facilitate learning, reflecting on innovation (#20, + 3*) is most valuable. *‘That you know where you want to go as an organization and in what way’* (respondent 26, staff member). Likewise, respondents mentioned the importance of collaborating with external partners on innovation themes (#19,+2*) and monitoring national innovation developments and trends (#18,+1*). To enable these actions, respondents indicate innovation budget availability as conditional for innovation readiness (#4, + 3*) *‘without time*,* space and resources*,* little happens’* (respondent 12, academic). Similarly to respondents holding perspective 1, they consider that (top)management’s role is paramount in indicating the organization’s innovation ambition, priorities, and route towards it (#27, + 4). *‘The board of directors has to be intimately involved in innovating’* (respondent 22). In line with perspectives 2 and 3 the respondent’s perspective of the role of the board and senior management is to encourage middle management to give space to and stimulate employees *‘to have the courage to experiment’* (#33, + 3) (respondent 8, (top)management).

Respondents suppose that employees, including middle management, in long-term care might not have innovating routinely high on their agenda and first have to be convinced to innovate before they are presented with knowledge for their role in innovating (#23,−4*). *‘I believe in a clear vision and direction*,* then enthusiastic people are eager to join in’* (respondent 8, (top)management). Respondents indicate that the role and tasks of employees (including middle management) while innovating should not be set in stone (#6,−4*) (#24,−3*). *‘Role clarity for employees will follow in time*,* enthusiasm is what you are looking for’* (respondent 22, (top)management). They favor encouraging employees to innovate relative to precise innovation instructions. *‘Approach it a bit more organically*,* when it comes to employee participation’* (respondent 12, academic). Comparably to those holding perspectives 1, 2, and 4, they do not consider the presence of innovation spaces important (#31, −4*). *‘People have to do it*,* availability of innovation spaces is not key’* (respondent 27, academic).

### Perspective 4: structuring decision-making, roles and responsibilities

Central to this perspective is respondents’ opinion that, to become innovation ready, an organization should formulate the innovation ambition (#1, + 4) and themes (#2,+4*) and organize the innovation organization accordingly (#27,+3) (#24,+3) (#6,+3*) (#11,+2*). *‘Formulating the ambition makes the intention of innovation concrete for employees: why do we want it?’* (respondent 18, academic). In line with perspective 2, the respondents of this perspective state that innovation must be linked to the strategy. *‘Innovation itself is no aim but a means to providing valuable and affordable healthcare*,* linked to the overall strategy of the organization’* (respondent 7, consultant). Furthermore, they point out that the innovation themes (#2,+4*) help to make deliberate choices that fit the ambition and character of the organization. Respondents see decision-making in the innovation process as conditional at all stages (#11,+2*) and ranked this statement more important than in perspectives 1,2 and 3. *‘You have to organize decision making otherwise you cannot take steps. That is why the innovation themes are so relevant. Participating in everything does not lead to success.*’ (respondent 3, innovation manager). In line with perspectives 1 and 3, the respondents express that the board plays a pivotal role in communicating the strategic innovation course (# 27,+3). *’The board must give direction and facilitate towards middle management and employees and communicate about the innovation direction and invite employees to engage’* (respondent 28, client representative). Notable in this regard is the respondent’s perspective that organizations should organize their own (innovation) course and not be dependent on innovation developments defined by others (statement 18, −3). *‘You should certainly monitor (inter-national) innovation developments*,* but you have to start with your own ambition and focus on ‘what does innovation X or Y* contribute’ (respondent 23, innovation manager).

Respondents in perspective 4 rank organizing the roles and tasks for middle management and employees (#24,+3) (#6,+3*) highest compared to perspectives 1,2, and 3. Respondents mention the importance of a clear innovation role of middle management, as they are seen as *‘the hub between care delivery and the board’* (#24,+3)’. *‘Middle management is the turntable*,* they form the connection between the top and other parts of the organization’* (respondent 17, consultant). Being clear about expectations and making agreements about the position and tasks of employees (#6,+3*) is seen as vital by the respondents as they are the ones performing the day-to-day activities in the organization and therefore paramount in achieving innovation results. *‘It is clear that healthcare professionals and staff must be given time and space to work on innovation otherwise they can not make the necessary innovation steps’* (respondent 17, consultant).

Respondents consider learning for innovation not as a one-off activity but as an important aspect that gradually takes place during the innovation process. Therefore, they rank statements such as ‘make innovation knowledge (gained in projects) available’ (#9,−4*) and ‘capture and evaluate learning experiences around innovation’ (#30,−3*) as less important. Furthermore, they prefer ‘to learn from success’ (instead of failure (#34,−3*) and ‘to copy experiences from other organizations’.

## Discussion

This study aimed to gain a deeper understanding of factors enabling innovation readiness in long-term care. Respondents agreed that the 36 statements (Table 1) represent a complete overview of factors contributing to innovation readiness of long-term care organizations for older adults. Respondents indicated that all 36 factors mattered at least to some extent for organizations to become better at innovating and that no important factors were missing from the set of statements. This also reaffirms the comprehensiveness of the framework outlining factors contributing to innovation readiness proposed in earlier studies on innovation readiness [36]. These findings are in line with the literature about innovation readiness in business [62, 63] and healthcare [64, 65].

Using these materials, we identified four distinct perspectives among stakeholders, each highlighting specific innovation readiness factors that are considered ‘most important’ within the context of long-term care for adults in the Netherlands: (1) ‘supportive role of management’ (2) ‘participation of the client (system) and employees’ (3) ‘setting the course and creating conditions’ and (4) ‘structuring decision-making, roles and responsibilities’. Perspectives 1, 3, and 4 are most aligned with the main factors ‘strategic course for innovation’ and ‘leadership for innovation’ of the innovation readiness framework [36] and indicate that these factors are considered central to innovation readiness. In line with this reasoning, respondents indicated that factors such as formulating an innovation ambition, providing an innovation budget, and decision-making should be seen as conditional.

In the post-sorting interviews, respondents added a developmental perspective to innovation readiness as they suggested there is a temporal order of the innovation readiness factors [66]. Several respondents advised following the ‘why’, ‘how’, and ‘what’ questions as a sequence for the importance of innovation readiness factors [67, 68]. The ‘why’ question, according to Sinek [68], is meant to align innovation goals with the purpose of the organization. Respondents indicated that an organization preferably starts with formulating its innovation ambition as it defines the intended reach of the innovation activities and guides decision-making concerning the choice of innovation projects. Payne et al. [69] explain the ‘how’ in innovation as the development of the skills, processes, and approaches to turn ambition into reality. The ‘what’ of innovation concerns innovation initiatives that are in line with the organization’s ambition [62].

The variety in what is considered important according to the four perspectives implies that there may not be a fixed route toward innovation readiness. Although a shared understanding of factors enabling innovation readiness resulted from the study, we must recognize that there is a nuanced and varied landscape of opinions among respondents when it comes to their perspectives on how to become innovation ready. Therefore, the internal alignment of stakeholders on innovation readiness will likely determine the most suitable route for the organization to become better at innovating [50]. This is in line with the volatile and multi-faced context of long-term care organizations [35] in which innovation is strongly influenced by and dependent on government policies [38], organizational conditions such as size and age of the organization [70] and characteristics of the innovation organization [9, 71].

Three perspectives (1, 3, and 4) stated a top-down perspective by expressing the importance of formulating innovation ambition (#1) and the pivotal role of management (#27) herein. Perspective 2 stated a more bottom-up perspective on innovation readiness by expressing weight on the involvement of healthcare professionals and the client system (#15, #16). Herewith, the role of middle managers is expressed as vital by the respondents as they have a pivotal role in both bottom-up and top-down as also shown by Birken et al. [72] and Urquhart et al. [73]. At the same time, respondents indicated that the organization has to be aware of the split roles of middle managers.

Conflicting situations might arise when managers might not be in favor of a proposed innovation, while at the same time, they are expected to shape the actions of individuals or teams in line with senior management plans. The same applies if the organizational culture acts as a potential constraint on the innovative efforts of managers [74, 75]. Furthermore, middle management has traditionally been trained to take care of day-to-day business and is not necessarily competent and trained to navigate innovation [76, 77]. Therefore middle management should be facilitated to fulfill their important role via sufficient resources and support [73, 78]. The role of the board and senior management is to encourage middle management to give space to and stimulate employees to have the courage to experiment [78, 79].

The respondents’ hierarchical position in the organization or professional role in long-term care did not seem to determine their perspective on factors enabling innovation readiness. Respondents from the six groups included in this study were associated with all four perspectives on innovation readiness, except for perspective 2 (participation of the client (system) and employees). Four of the five respondents associated with this perspective had a similar role in long-term care: representing clients to ensure that their viewpoints are heard and reflected in organization policies. Perspective 1 (supportive role of management) opposes perspective 2 in arguing that although it is desirable to give clients and their families a voice while innovating, they should only be involved when it is seen as relevant to their situation by management. Resident advisory councils seem to balance these perspectives (1 and 2) as only a few councils exercise their legal right to be consulted for organizational issues like innovating [80].

This study identifies and provides a valuable overview of innovation readiness factors that enhance the ability to innovate effectively for long-term care organizations for older adults. Organizations that prioritize these areas might be able to better navigate their innovation challenges. Future research could focus on the development of an assessment tool derived from the innovation readiness statements presented in this study. Such a tool would facilitate an assessment of the innovation maturity of long-term care organizations for older adults and identify opportunities for enhancing innovation readiness.

### Strengths and limitations of this study

Several strengths and limitations of this study should be considered. First, our study was conducted in the long-term care sector in the Netherlands. The identified perspectives may, therefore, not represent the perspectives of respondents in other healthcare sectors and organizations in the Netherlands, or healthcare organizations outside of the Netherlands. Second, the four perspectives together explained 46% of the variance in the rankings of the statements by respondents. Although representing a frequently occurring percentage in Q-methodology studies [42], meaning that the perspectives capture significant shared perspectives on innovation readiness, there still may be more nuance to these perspectives in practice. However, more importantly, respondents indicated all factors of relevance to be included in the statement set and, hence, to be able to share their perspective adequately through the materials. Finally, the Q-methodology studies are intended to be an exploratory tool, providing insight into the heterogeneity of views on a specific topic [41]. Nevertheless, there is no certainty whether the selection of respondents captures all relevant variation. The results of this study show four relevant perspectives but do not indicate how prevalent they are and among which stakeholders. Further research is needed in this regard. A strength of the study is that we collected rich quantitative and qualitative data that together allowed for an in-depth investigation into the variety of perspectives on innovation readiness in long-term care. The sample of respondents recruited for this study meets the choices of the number of participants of the Q-methodology [40, 81] and is similar to those of other studies [42]. The use of online software to perform the ranking of the statements instead of manually sorting cards saved time and eliminated the need to manually enter the data [82, 83]. The interviewer could follow in real-time the card sorting and comments made by the respondent. Although face-to-face interviews in the work setting of the respondent might have brought more in-depth information versus conducting online card-sorting and interviews. Furthermore, online card sorting and the accompanying post-interviews gave the possibility to have a sample with a wide geographical distribution and lower costs of administration. Finally, the materials developed can effortlessly be used to replicate this study in other healthcare sectors such as hospitals and welfare, although conducting a pilot study to check the comprehensiveness and clarity of the statement set in each contact is recommended.

## Conclusions

The shared understanding of factors enabling innovation readiness reaffirmed the evidence-based framework of innovation readiness factors of long-term care organizations for older adults [36]. The main factors ‘strategic course for innovation’ and ‘leadership for innovation’ are central to innovation readiness. Furthermore, the comprehensiveness of the list of factors contributing to innovation readiness [36] was endorsed. The heterogeneity in stakeholders’ perspectives shows a nuanced landscape of opinions toward becoming better at innovating. This study’s results indicate that becoming innovation ready requires deliberate preparation upfront such as strategy, time, financial resources, and expertise that are not always readily available within a specific single innovation initiative. Research into innovation readiness of healthcare organizations is a rather new field. This research shows which innovation factors are important for innovation readiness, rather than how these factors can contribute to innovation readiness. The tendency to perceive innovation readiness as an evolving process (as mentioned by the respondents) could help to understand and explain how innovation readiness can be nurtured and grown over time. Furthermore, future research could be directed toward developing an asessement tool, based on this study’s innovation readiness statements, assessing the maturity of long-term organizations for older adults and providing direction to opportunities for innovation readiness.

## Supplementary Information

Additional file 1.

Additional file 2.

Additional file 3.

Additional file 4.

Additional file 5.

Additional file 6.

Additional file 7.

## Data Availability

The data that support the findings of this review are included in this published article and are openly available in Open Science Framework at https://osf.io/h7tfp/.

## References

[1] Barber SL, Van Gool K, Wise S, Wood M, Or Z, Penneau A, Lorenzoni L. Pricing long-term care for older persons. In Pricing long-term care for older persons. 2021

[2] Institute NHC. The Dutch health care system 2024 [cited 2024 Sept 4]. https://english.zorginstituutnederland.nl/about-us/healthcare-in-the-netherlands.

[3] Verbeek FHO, Van Lierop MEA, Meijers JMM, Van Rossum E, Zwakhalen SMG, Laurant MGH, et al. Facilitators for developing an interprofessional learning culture in nursing homes: a scoping review. BMC Health Serv Res. 2023;23(1):Facilitators for developing an interprofessional learning culture in nursing homes: a scoping review. 10.1186/s12913-023-09092-5 PMC9945386 36810021

[4] Chaves BBC, Bouabida K. Innovation in Healthcare Organizations: Concepts and Challenges to Consider. Int J Health Res Innov. 2021;9:1–14.

[5] Lyng HBRE, Wibe T, Wiig S. Healthcare leaders’ use of innovative solutions to ensure resilience in healthcare during the Covid-19 pandemic: a qualitative study in Norwegian nursing homes and home care services. BMC Health Serv Res. 2021;21(1):878. 34446000 10.1186/s12913-021-06923-1 PMC8390181

[6] World Health Organization. Framework for countries to achieve an integrated continuum of long-term care. World Health Organization. 2021. https://iris.who.int/handle/10665/349911.

[7] Kievit PJOJ, Schoorl M, Bartels P. The missing link: toward an assessment of innovation capacity in health care organizations. Int J Qual Innov. 2018;4:1–18.

[8] Williams I. Organizational readiness for innovation in health care: some lessons from the recent literature. Health Serv Manage Res. 2011;24(4):213–8. 22040949 10.1258/hsmr.2011.011014

[9] Greenhalgh TRG, Macfarlane F, Bate P, Kyriakidou O. Diffusion of innovations in service organizations: systematic review and recommendations. Milbank Q. 2004;82(4):581–629. 15595944 10.1111/j.0887-378X.2004.00325.x PMC2690184

[10] Manly J, Ringel M, MacDougall A, Cornock W, Harnoss J, Apostolatos K, Izaret JM. Reaching New Heights in Uncertain Times. Boston: Boston Consulting Group; 2023.

[11] Lokuge SSD, Grover V, Xu DM. Organizational readiness for digital innovation: development and empirical calibration of a construct. Inf Manag. 2019;56(3):445–61.

[12] Van Den Hoed MW, Backhaus R, De Vries E, Hamers JPH, Daniëls R. Factors contributing to innovation readiness in health care organizations: a scoping review. BMC Health Serv Res. 2022;22(1):997. 35932012 10.1186/s12913-022-08185-x PMC9354428

[13] Nolte E, World Health Organization. How do we ensure that innovation in health service delivery and organization is implemented, sustained and spread?. 2018.

[14] Casanova G, Principi A, Lamura G. Social Innovation in Long-Term Care: lessons from the Italian case. Int J Environ Res Public Health. 2020;17(7):2367. 32244446 10.3390/ijerph17072367 PMC7177354

[15] Collaborating Academic Networks for Elder Care Netherlands. Knowledge Agenda long-term care for older adults. Collaborating Academic Networks for Elder Care Netherlands; 2023.

[16] Dutch Ministry of Health Welfare and Sport Integral Care. Integral Care Agreement 2022 Working together for healthy care. 2022.

[17] Weatherford B, Bower KA, Vitello-Cicciu J. The CNO and leading Innovation: competencies for the future. Nurs Adm Q. 2018;42(1):76–82. 29194335 10.1097/NAQ.0000000000000263

[18] Anvik C, Vedeler JS, Wegener C, Slettebo A, Odegard A. Practice-based learning and innovation in nursing homes. J Workplace Learn. 2020;32:122–34.

[19] Atkinson MK, Singer SJ. Managing Organizational Constraints in Innovation Teams: A Qualitative Study Across Four Health Systems. Med Care Res Rev. 2021;78(5):521-36. 10.1177/1077558720925993. 10.1177/1077558720925993 PMC8483614 32552540

[20] Hunter RB, Winston FK, Dehel P, Oh K, Nicklas J, Hartung H. SPRINTing to Innovation: children’s hospital of Philadelphia’s Strategic Approach to discovering its untapped Innovation potential. Acad Med. 2021;96(4):534–9. 33208677 10.1097/ACM.0000000000003852

[21] Hyrkas PHL, Vainamo S, Iivari M, Sachinopoulou A, Majava J. Collaborative innovation in healthcare: a case study of hospitals as innovation platforms. Int J Value Chain Manag. 2020;11(1):24–41.

[22] Jaskyte K, Dressler WW. Organizational culture and innovation in nonprofit human service organizations. Adm Soc Work. 2005;29(2):23–41.

[23] Leal-Rodríguez ALRJ, Leal AG, Ortega-Gutiérrez J. Knowledge management, relational learning, and the effectiveness of innovation outcomes. Serv Ind J. 2013;33(13):1294–311.

[24] Lombardi MM, Spratling RG, Pan W, Shapiro SE. Measuring Organizational Capacity To Accelerate Health Care Innovation in Academic Health Centers. Qual Manag Health Care. 2018;27(1):1–7. 29280901 10.1097/QMH.0000000000000157

[25] Barnett J, Vasileiou K, Djemil F, Brooks L, Young T. Understanding innovators’ experiences of barriers and facilitators in implementation and diffusion of healthcare service innovations: a qualitative study. BMC Health Serv Res. 2011;11:342. 22176739 10.1186/1472-6963-11-342 PMC3265424

[26] Zippel-Schultz BSC. Mediated and moderated effects of business and project planning on innovation projects in hospitals. Creat Innov Manag. 2011;20(4):296–310.

[27] Dohan MSGM, Tan J. The impact of healthcare informatics competencies on dynamic capabilities: a multilevel study of paramedic services. Health Policy Technol. 2017;6(4):426–35.

[28] Emiralioglu R, Sonmez B. The relationship of nursing work environment and innovation support with nurses’ innovative behaviours and outputs. J Nurs Manag. 2021;29(7):2132–41. 33930243 10.1111/jonm.13354

[29] Glover WJ, Nissinboim N, Naveh E. Examining innovation in hospital units: a complex adaptive systems approach. BMC Health Serv Res. 2020;20(1):554. 32552869 10.1186/s12913-020-05403-2 PMC7302354

[30] Renkema MdLJ, Van Zyl LE. High-involvement HRM and innovative behaviour: the mediating roles of nursing staff’s autonomy and affective commitment. J Nurs Manag. 2021;29(8):2499–514. 34062030 10.1111/jonm.13390 PMC8596638

[31] Nieboer AP, Strating MM. Innovative culture in long-term care settings: the influence of organizational characteristics. Health Care Manage Rev. 2012;37(2):165–74. 21720249 10.1097/HMR.0b013e318222416b

[32] Zuber CD, Moody L. Creativity and Innovation in Health Care: tapping into organizational enablers through human-centered design. Nurs Adm Q. 2018;42(1):62–75. 29194334 10.1097/NAQ.0000000000000267

[33] Jonsson TFUC, Kähler HG. Do autonomous and trusting hospital employees generate, promote and implement more ideas? The role of distributed leadership agency. Eur J Innov Manag. 2022;25(1):55–72.

[34] Thoma - Lürken T. Innovating long-term care for older people: development and evaluation of a decision support app for formal caregivers in community-based dementia care. Maastricht: Datawyse / Universitaire Pers Maastricht. 2018:169 p. 10.26481/dis.20180919ttl.

[35] Rycroft-Malone J, Rogers L, Burton CR. Optimising the Conceptualisation of Context; comment on stakeholder perspectives of attributes and features of Context relevant to knowledge translation in Health settings: a multi-country analysis. Int J Health Policy Manag. 2022;11(10):2365–7. 37579347 10.34172/ijhpm.2022.6900 PMC9808282

[36] Van Den Hoed MW, Backhaus R, Beaulen A, Hamers JPH, Daniels R. Factors enabling innovation readiness of long-term care organizations: stakeholder opinions. PLoS One. 2024. Submitted for publication.

[37] Hardy MS, Sasseville M, Attieh R, Bergeron-Drolet LA, Sanchez RHB, Gallani MC, et al. Assessing facilitating conditions and barriers for innovation implementation in Canadian long-term care homes: a research protocol. Implement Sci Comm. 2022;3(1):61. 10.1186/s43058-022-00312-3 PMC9187889 35690855

[38] Jongen W. The impact of the long-term care reform in the Netherlands: an accompanying analysis of an ‘ongoing’ reform. Maastricht: Datawyse / Universitaire Pers Maastricht. 2017:205. 10.26481/dis.20170223wj.

[39] Marjanovic SAM, Hocking L, Chataway J, Ling T. Innovating for improved healthcare: Sociotechnical and innovation systems perspectives and lessons from the NHS. Sci Public Policy. 2020;47(2):283–97.

[40] Watts SSP. Doing Q Methodological Research: Theory, Method and Interpretation. London. 2012.

[41] Churruca K, Ludlow K, Wu W, Gibbons K, Nguyen HM, Ellis LA, et al. A scoping review of Q-methodology in healthcare research. BMC Med Res Methodol. 2021;21(1):125. 34154566 10.1186/s12874-021-01309-7 PMC8215808

[42] Dieteren CMPN, Reckers-Droog VT, van Exel J. Methodological choices in applications of Q methodology: a systematic literature review. Soc Sci Humanit Open. 2023;7(1):100404.

[43] Krause KRE-CJ, Bear HA, Calderón A, Wolpert M. What treatment outcomes matter most? A Q-study of outcome priority profiles among youth with lived experience of depression. Eur Child Adolesc Psychiatry. 2023;32(1):123–37. 34273026 10.1007/s00787-021-01839-x PMC9908724

[44] Van Exel JDGG. Q methodology: A sneak preview 2005. https://scholar.google.nl/scholar?oi=bibs&cluster=13840018368612320153&btnI=1&hl=en.

[45] ICT&Health. ICT&Health 2021–2023. https://icthealth.nl/magazine/.

[46] Zorgvisie. Zorgvisie magazine: Zorgvisie magazine; 2021–2023. https://www.zorgvisie.nl/magazine/category/zorgvisie-magazine/.

[47] Watts S, Stenner P, Doing. Q Methodological Research: Theory, Method and Interpretation. London. 2012 2023/02/03.

[48] Paige JB, Morin KH. Q-Sample Construction. A critical step for a Q-Methodological study. West J Nurs Res. 2016;38(1):96–110. 25092207 10.1177/0193945914545177

[49] Greenhalgh T, Papoutsi C. Spreading and scaling up innovation and improvement. BMJ. 2019;365:l2068. 31076440 10.1136/bmj.l2068 PMC6519511

[50] Mintzberg H. Understanding Organizations... Finally!: Structuring in Sevens. Berrett-Koehler Publishers. 2023.

[51] Heidari S, Babor TF, De Castro P, Tort S, Curno M. Sex and gender equity in Research: rationale for the SAGER guidelines and recommended use. Res Integr Peer Rev. 2016;1:2. 29451543 10.1186/s41073-016-0007-6 PMC5793986

[52] Moser A, Korstjens I. Series: Practical guidance to qualitative research. Part 3: Sampling, data collection and analysis. Eur J Gen Pract. 2018;24(1):9–18. 29199486 10.1080/13814788.2017.1375091 PMC5774281

[53] Lutfallah SBL. Quantifying subjective data using online Q-methodology software. Volume 14. The Mental Lexicon; 2019.

[54] Howlett M. Looking at the ‘field’ through a zoom lens: methodological reflections on conducting online research during a global pandemic. Qualitative Res. 2022;22(3):387–402. 10.1177/1468794120985691 PMC9095994 35663097

[55] Shemmings DET. Using Q methodology in qualitative interviews. The SAGE handbook of interview research: The complexity of the craft. 2012;2:415–26.

[56] Legard RKJ, Ward K. In-depth interviews. In: Ritchie JLJ, editor. Qualitative research practice: A guide for social science students and researchers, vol. 1. Sage; 2003.

[57] Korstjens I, Moser A. Series: Practical guidance to qualitative research. Part 2: context, research questions and designs. Eur J Gen Pract. 2017;23(1):274–9. 29185826 10.1080/13814788.2017.1375090 PMC8816399

[58] Raskind IG, Shelton RC, Comeau DL, Cooper HLF, Griffith DM, Kegler MC. A Review of Qualitative Data Analysis Practices in Health Education and Health Behavior Research. Health Educ Behav. 2019;46(1):32–9. 30227078 10.1177/1090198118795019 PMC6386595

[59] Akhtar-Danesh N. Impact of factor rotation on Q-methodology analysis. PLoS ONE. 2023;18(9):e0290728. 37656676 10.1371/journal.pone.0290728 PMC10473483

[60] Brown S. Political subjectivity: applications of Q methodology in political science. Yale University Press; 1980;2:262−74.

[61] KADE. KADE. https://github.com/shawnbanasick/kade.

[62] Tidd JBJ. Managing innovation: integrating technological, market and organizational change. Hoboken, NJ, USA. Wiley; 2020.

[63] Hinsch M. ISO 9001: 2015 for Everyday operations: all facts–short. Concise and Understandable: Springer; 2019.

[64] Nauta F, Crombach M. Dutch healthcare innovation scan: An inventory of innovation management in the Dutch healthcare sector. 2011.

[65] Fleuren M, Wiefferink K, Paulussen T. Measurement instrument for determinants of innovations (MIDI). Int J Qual Health Care. 2014;16(2):107–23. 10.1093/intqhc/mzu060 PMC4195468 24951511

[66] Nilsen P. Making sense of implementation theories, models, and frameworks. Implement Sci 302020. pp. 53–79. 10.1186/s13012-015-0242-0 PMC4406164 25895742

[67] Hamel G. The why, what, and how of management innovation. Harvard Bus Rev. 2006;84(2):72–84. 16485806

[68] Sinek S. Start with why: how great leaders inspire everyone to take action. Penguin; 2009;1:1–245.

[69] Payne M. How to kill a unicorn: How the world’s hottest innovation factory builds bold ideas that make it to market. Crown Business; 2014;10:1–304.

[70] Damanpour FSM. Phases of the Adoption of Innovation in Organizations: effects of Environment, Organization and top managers. Br J Manag. 2006;17(3):215–36.

[71] Damschroder LJRC, Widerquist MAO, Lowery J. The updated Consolidated Framework for Implementation Research based on user feedback. Implement Sci. 2022;17(1):75. 36309746 10.1186/s13012-022-01245-0 PMC9617234

[72] Birken SA, DiMartino LD, Kirk MA, Lee SY, McClelland M, Albert NM. Elaborating on theory with middle managers’ experience implementing healthcare innovations in practice. Implement Sci. 2016;11:2. 26729367 10.1186/s13012-015-0362-6 PMC4700583

[73] Urquhart R, Kendell C, Folkes A, Reiman T, Grunfeld E, Porter GA. Making it happen: middle managers’ roles in Innovation implementation in Health Care. Worldviews Evid Based Nurs. 2018;15(6):414–23. 30291739 10.1111/wvn.12324 PMC6518932

[74] Øvretveit J, Andreen-Sachs M, Carlsson J, Gustafsson H, Hansson J, Keller C, et al. Implementing organisation and management innovations in Swedish healthcare: lessons from a comparison of 12 cases. J Health Organ Manag. 2012;26(2):237–57. 22856178 10.1108/14777261211230790

[75] Burgess NCG. The knowledge brokering role of the hybrid middle level manager: the case of healthcare. Br J Manag. 2013;24:132–42.

[76] Birken SA, Lee S-YD, Weiner BJ. Uncovering middle managers’ role in healthcare innovation implementation. Implement Sci. 2012;7(1):28. 22472001 10.1186/1748-5908-7-28 PMC3372435

[77] Oldenhof L. The multiple middle: managing in healthcare. Erasmus University; 2015.

[78] Birken SA, Lee SY, Weiner BJ, Chin MH, Chiu M, Schaefer CT. From strategy to action: how top managers’ support increases middle managers’ commitment to innovation implementation in health care organizations. Health Care Manage Rev. 2015;40(2):159–68. 24566252 10.1097/HMR.0000000000000018 PMC4141032

[79] Engle RLLE, Gormley KE, Chan JA, Charns MP, Lukas CV. What roles do middle managers play in implementation of innovative practices? Health Care Manag Rev. 2017;42(1):14–27. 10.1097/HMR.0000000000000090 PMC5131695 26488239

[80] Zuidgeest MLK, Westert GP, Delnoij DM. Legal rights of client councils and their role in policy of long-term care organisations in the Netherlands. BMC Health Serv Res. 2011;11(1):215. 21910899 10.1186/1472-6963-11-215 PMC3181203

[81] Brown S. Q methodology and qualitative research. Qual Health Res. 1996;6(4):561–7.

[82] Alanazi AS, Wharrad H, Moffatt F, Taylor M, Ladan M. Q methodology in the COVID-19 era. Healthcare. 2021;9(11):1491. 34828537 10.3390/healthcare9111491 PMC8620337

[83] Meehan K, Ginart L, Ormerod KJ. Short take: sorting at a Distance: Q methodology online. Field Methods. 2022;34(1):82–8.

