# Supplementary material for: Perspectives on managing innovation readiness in long-term care: a Q-methodology study
Source: BMC Geriatr. 2024 Dec 19;24:1017. doi: 10.1186/s12877-024-05572-3 (PMC11658053; doi:10.1186/s12877-024-05572-3)
Supplement: Supplementary file 1 — Additional file 1. [file 12877_2024_5572_MOESM1_ESM.docx]

**Qstudy IR answers additional factors**

Q1: No further discussion needed.

Q2: No further discussed needed.

Q3: Provide room for experimentation.

Q4: Much recognition.

Q5: Two things seem less relevant; they appear to be of a different order.

Q6: He is not sure. He would need to compare it with ‘Obstacles,’ his article on structural innovation.

Q7: Fun/enjoyment/humor.

Q8: Included: positioning middle management.

Q9: Willingness to change (personal factor)

Q10: No omissions, seems quite complete in relation to practice.

Q10: Sufficient budget for investment (as mentioned by the respondent).

Q11: Innovation objectives (slightly more specific than innovation ambition) should be clearly defined for each project.

Q12: The term “employees” is general. There is a significant difference between involving professionals like doctors versus staff.

Q13: Yes, there is an overlap in factors.

Q14: Not discussed.

Q15: No further discussion needed

Q16: Provide room for experimentation.

Q17: This is complete.

Q18: Organizations often hide behind laws, regulations, and quality standards, saying ‘we would like to, but it is not allowed.

Q19: Support and commitment within your organization are crucial.

Q20: No further discussion needed

Q21: When implementing technology, first consider how it can add value to your organization’s issues. Align the innovation process accordingly and select appropriate technology based on this. This is related to factor 16.

Q22: No further discussion needed

Q23: Innovation themes within your organization are closely tied to your strategy.

Q24: Yes, there are good points.

Q25: She believes the factors form good clusters, especially regarding roles, tasks, and organizational processes.

Q26: No further discussion needed

Q27: Informal leaders (who communicate with more people and have more influence) are more important than formal leaders. ‘You need to know them but involve them differently. Friction is necessary to develop a good innovation plan.

Q28: Co-creation with a supplier, depending on whether it is new to the market or the organization.

Q29: Concerns general staff, healthcare workers, and middle management.

Q30: No further discussion needed

Q1

Q1 1 Formulate inn amb idem 27

Q1 3 Describe what inn is idem 27

Q1 5 Make an MJP for inn idem 27

Q1 6 Internal development costs = time for tasks should not be forgotten in new projects. Share with teammates, others take over my work, invest time for focused work consultation. Am I willing as an organization to pay for this time.54

Q1 15 It is the people who have to do it. Do it together, therefore important that family and loved ones are involved in innovation. You do it for the quality of care for the person who needs care. Which gets its value from the contacts someone has with their family and loved ones.

Q1 16 Work floor, hands at the bedside, have innovation readiness become part of the toolbox. That's where the essences of innovation readiness are (34:26)

Q1 19 Collaboration with external partners, also applies to other domains like welfare. You have to be an open organization VVT should not be a bubble1.0522

Q1 21 Education focused on learning to innovate, difficult, 55.24 rather let team determine what they need from the question, what do you need to do your job. A central question that should be asked continuously. 56.48

Q1 22 Inn teams int disc compose idem 27

Q1 23 Clear role on innovation for middle mng

Q1 24 middle management makes translation from strategy to floor practice 43.10 and needs knowledge and support in doing so.44.15

Q1 25 It's the employees who have to do it. Organization must value what employees contribute to innovation. Not just salary, commitment from employees that that is seen and named and becomes a permanent part of their performance e.g. in performance reviews. This is important to get innovation off the ground on the shop floor. 38.17

Q1 27 Board of Directors carries from the inn an org, prio is. Clearly the least important. Innovation is a means and not an end. If you make innovation an organizational priority then it becomes an objective.1.01

Q1 30 Capture and evaluate learning experiences around innovation you can do that both horizontally and vertically 51 strat-tac-operational or departments among themselves. (Exactly on the middle of the grid). Central: what do you need

Q1 33 Having guts is only possible in a safe environment where things are allowed to go wrong. If there is appreciation then there will also be room to be bold. 40.10

Q1 34 See learning from mistakes made very broadly oa communication plan for innovation needed, in which learning and evaluation plays an important role. In interaction between management and shop floor, make the journey together and discover together. Bv stop and that also takes guts. 50 Communication must start with the shop floor, because they can give back experiences. 49

Q1 35 Taking time to learn, because it's about behavior change. 40.35 and that takes a lot of time. How do you get to a new routine. That's where you have to start. In the end, people make the innovation. Metaphor: lace up shoes differently and see how long that takes.

Q1 26/28/32 Middle manager has to encourage employees, tell them what the role is (and that requires something different with each employee) and that it is nice to be involved 46

Q2

Q2 1 17.59 formulate innovation ambition. Shared vision. Creative climate. Have organization in order.

Q2 18 monitor national innovation developments least important. In the organization it has to be in order first, if you want to go outside afterwards. Already happening as a matter of course. E.g. through Linkedin, ICT&Health. You already do that continuously. Less important to become innovation ready.

Q2 What is already happening in your own organization plays an important role in this.

Q2 Employees do not know of themselves how 'innovative' they are sometimes already doing. E.g. making smaller laundry cart because it is too heavy otherwise. Small incremental type 1 innovations, important to make employees aware of this. Awareness is part of innovation readiness.

Q2 Opinion: Too much emphasis on technological innovation (also from the government). Too little on process and social innovations, but necessary for sustainable innovation e.g. dinamo model (focused on change model).

Q2 Organization consists of many small sub-organizations, IR at site level, department level? Is there a common thread there? In middle management attention to leadership. With employees e.g. attention to innovative competencies.

Q2 27 Management conveys that innovation is an organizational priority. Leadership needed to get employees on board in terms of willingness to change with a clear story. Also to enthuse middle management.

Q2 24 Middle management support is so important, especially in time of scarcity. Employees are mostly at the bedside. Getting employees into a project group is difficult but is important to change with them.

Q2 23 Innovation board gets middle management involved with shop floor. To get to work together.

Q2 26 Available innovation hours are often scheduled for daily care work. Middle management sits between care and board. To make connection between upstairs and downstairs: the hub 31. Important to know if a site or department is IR. Middle management knows what is going on on the shop floor, what the wishes are in/to solve problems on the shop floor. And they ideally oversee the bigger picture and estimate whether innovations fit/live in the team.

Q2 19 Collaborating with external partners. If you want to start collaborating you have to get it right internally first. 23.08 Otherwise, what is collaboration worth if you don't know if you can implement it properly

Q2 33 Having guts to experiment, that's natural. Innovating is off the beaten path anyway, you're already showing guts. You don't make the organization more innovation ready with that. 25.10

Q2 15 Involve family and loved ones, client system very important, also causes noise on the line, involve must, but you must first get it right yourself. Involve at a later stage.

Q2 17 Share knowledge yes, but first get it in order internally.

Q3

Q3 1 Start point to be engaged in innovation for organization and cooperation between organizations. Is very diverse in the region. Is helpful in making choices. And for people in the organization to understand why choices are made and what it is based on. You have to make this ambition communicable. An 80-page plan is not going to work. It goes beyond formulating an ambition also spreading it.

Q3 5 Does not believe in establishing a multi-year plan for innovation. This is at the expense of the organization's agility16.35

Q3

Q3 Have time resources space to innovate, to be involved in it, it is a continuous process, for every discipline in the organization. 25.49 End result you are working on it daily changing , improving, that it comes into the personal leadership of every employee. Collective action perspective on innovation 26.38 & 38.05

Q3 In the regional project there is a lot of focus on learning from each other on learning to innovate. 23.51 Especially to get rid of the syndrome in elder care that you want to invent everything yourself and do research because only then is it the truth. And continuing to develop from collaboration. The whole of moving with each other leads to further development and success with each other.

Q3 All forms of innovation require attention to social processes 51.00

Q3 This requires getting to know each other and seeing each other through the year. Whereby you look each other up and ask questions. Sharing has now become normal at Anders werken in de Zorg. 25.00

Q3 Anders werken in de zorg facilitates: e.g. make bus available for participating organization to go to ICThealth fair together and then pick up what top 3 important insights were and develop that further in the program of Anders werken in de zorg.

Q3 2 Establishing theme gives more breadth than a multi-year plan for innovation. And helps to make conscious choices that fit the ambition and character of the organization 17.00 Has relationship with decision-making.

Q3 11 Making agreements about decision making in decision making process is important in all phases of the process. Too anarchically organized then you can't take steps. Therefore in line with this the innovation themes are relevant. Not to choose something is also a choice. Taking part in everything does not lead to success

Q3 36 Sharing and exchanging knowledge regarding innovation important 9.15 especially because organizations want and need so much. You have to get rid of the idea that organizations individually reinvent things. You have to see it as a joint task. Part of having a vision of learning. The innovation plan should include a paragraph about how you deal with learning from innovation, related to work climate. Learning from what you are good at.

Q3 17 Knowledge sharing and exchange regarding innovation important 9.15 especially because organizations want and need so much. You have to get rid of the idea that organizations individually reinvent things. You have to see it as a joint task. Sharing innovation knowledge is important for further development in the field of innovation 34.27, collec

Q3 10 Conscious communication about innovation how do you communicate, terminology and when do you involve whom. To avoid innovation becoming some kind of bigbang. Bring employees into awareness so they will take initiative on innovation themselves. Communication continuously and from projects. Having a communication strategy is important.

Q3 24 Middle management role may get more attention19.27 first focused on care employees, and project leaders approached. But then you miss the interpretation. Projects need to go back to the line. Middle mng can facilitate like no other.

Q3 20 Vision on learning

Q3 28 Clear role for employees is already in 25

Q3 9 Is already covered in her eyes by giving attention to 36

Q3 19 Is already in 17

Q3 34 Learning from mistakes made, especially learning from success and how to copy that from each other.

Q3 Have made their own questionnaire about innovation maturity, based on literature (also based on inspiration session of March 2, 2023) And put it out and based on that they have developed a learning course because there was a need for that for managers and directors .1x per quarter they come together -> focused on awareness and dialogue and learning from each other again. This is supported and conceived by working differently in healthcare.

Q4

Q4 15 Not just involve in innovation process. You want an innovation because you think that's better for people who are getting the care and delivering that care and the network around it, so they have to do something different as well. That's the lowest form of participation. For different behavior, then you have to do more awareness raising, entice someone to start showing different behavior (quote).

Q4 31 Classroom helps, can inspire, doesn't say you shouldn't do it, you can manage without it too

Q4 16 Soft aspects of the organization matter and determine whether an innovation can be successful. Behavioral aspects of employees and elders (overcome: we already do that, that doesn't work) to avoid blocking innovation.

Q4 25 Include employees in that their work is changing, and ask for suggestions. You get task-oriented training. That process takes a lot of time. social components it's the people who have to do it.

Q4 27 Role of director, broad view of innovate (all types of innovation) and really want to innovate, not be like mandatory number

Q4 28 Bring employees along in that their work is changing, and ask for suggestions. You get task-oriented training. That process takes a lot of time.

Q4 8 As a counterbalance that's where a lot of attention is already going,

Q5

Q5 27 Organizations that are most successful in innovation are the most top-down organized, with a director who says this is how we are going to do it. You need vision and direction. A director can put an organization in the right position.

Q5 21 Education is important in innovation, in management. For care employees it makes sense to bring a practical innovation into the project/traject, so much more concrete approach

Q5 Respondent is convinced of the role of leadership (style), determines the innovation culture of an organization and how to organize it.

Q5 33 Guts, we tend to make a buttoned-up plan first, BC must be around, before you start implementing innovation try. Genius innovation come because you give room for trial and error. Exciting. Administrative vision needed for that and dot on the horizon. You get further ahead with that than just nailing everything down in advance. It takes guts of management, because they make the decision. QUOTE

Q5 16 Factors mixed. Started with management, that's where it starts, as management you can't say we're going to turn left. That's where you have to involve employees in that process. It doesn't start with the employee, it starts with the board. Employees determine whether something can be promising or successful. QUOTE

Q5 10 Communication. The plan can be so good, stay out carry how, why and what you are going to do and keep telling that continuously. You should not think that communication is something you can just throw in. Important for something to land in the organization.

Q5 30 Learning experiences, you want to know if something makes sense what you are doing. You want to know why something worked or didn't work. As long as you learn from it, it has been a meaningful innovation. Learning also depends on how innovation is embedded in the organization. Eg small organization where innovation is not really organized, fragmentation, that makes it difficult to store learning experiences and use them again. Organize a place to land learning experiences (from consultant role)

Q5 3 So do you need to define innovation? Innovation is a means to an end, with a logical strategy to achieve the end

Q5 31 Physical spaces, sometimes it is necessary, often less important, depends on the innovation most integrate into current work processes. So you don't need another space for that.

Q5 15 Depends on the type of innovation. There are also innovations where family and loved ones don't notice anything. If it has an impact then you have to inform people, but do you always have to involve like that? But this is not so accepted to say.

Q5 32 You don't want to discourage people from trying something out. If this is the strategy: to encourage employees to get started innovating, you're not going to make it. So therefore more to less important
