# Supplementary material for: Perspectives on managing innovation readiness in long-term care: a Q-methodology study
Source: BMC Geriatr. 2024 Dec 19;24:1017. doi: 10.1186/s12877-024-05572-3 (PMC11658053; doi:10.1186/s12877-024-05572-3)
Supplement: Supplementary file 2 — Additional file 2. [file 12877_2024_5572_MOESM2_ESM.docx]

Q6

Q6 5 Multi-year plan: Radical innovation is very exploratory in nature, so you can't look 5 years ahead. Setting a dot on the horizon in terms of criteria and principles can be done, but making a multi-year plan cannot.

Q6 8 Technical infrastructure: More supportive, but not decisive. Doesn't make the big difference. There is a lot of focus on technological innovation, when in fact that is not the biggest issue. It's much more about changing processes. Of course, it helps if your infrastructure is ready for this. The basics have to be in order (good wifi, for example). But the focus is sometimes a bit too much on this. Then you get: old process + expensive technology = expensive old process 01:01:00

Q6 22 Put together innovation team(s) interdisciplinary: Working interdisciplinary is incredibly important for innovation. You need different perspectives and different competencies when innovating, to get it done. Doesn't necessarily have to be an innovation team, but you have to do it multidisciplinary.

Q6 24 Clear role of middle management on innovation: Board is even more important than middle management. Middle management does determine the daily work of the care workers, but it is questionable how big their effects are with regard to innovating. However, it is important that they have a facilitating role in this. However, in innovation, the roles of everyone involved are often more fluid, they do not have to be so clearly defined.

Q6 27 Board conveys that innovation is an organizational priority: Board must give space so that people have time to engage in innovation. If not, innovation falls off the agenda first, because innovation is something that pays off indirectly or only in the longer term. Directors must therefore indicate that it is okay to do this. Because questions of: is this really necessary and what will it get us?

Q6 28 Clear role of employees in terms of innovation: Clear roles he values somewhat less. Employees can have different roles. If they have a good idea, let them participate in an innovation project. If someone doesn't have these skills, that's okay too. Framed roles are not necessary.

Q6 31 Physical spaces: See above (under Technical infrastructure)

Q6 34 Learning from mistakes made: see above

Q6 35 Taking time to learn: see above

Q6 36 Learning from each other in terms of innovating: These and the two factors below looked a bit like a cluster. A good example: there is always a lot of discussion about pilots: is this a good thing to do or not? If the technology is proven effective, then it is no longer so relevant for an organization to go pilots to further develop the technology, but rather to see how such an innovation works in the context of your organization. That is hugely important, to have those preconditions in place, and to be able to do it even better next time.

Q7

Q7 2 Innovation is not an end but a means to deliver good care. Using technology as a lever, and sometimes we call it innovation. Innovation must be linked to strategy. (If an implementation) of an innovation is linked to that then it goes much better 45.00.

Q7 4 Separate budget with that you set innovation apart, however in every budget there should be a pot of exploration. And that you have to explain if you haven't used this money. Rather: look at what is the problem, how big is this and what is its effect. And make a business case out of that. If you have a big enough problem to tackle, then the money will 'come naturally'. Eg voice-based reporting. A project leader must learn how to play this to bring others along in this story (time, express in concrete terms in money, what irritates, do the math, business case). Is tricky for both project leaders and staff though.

Q7 8 Basic infrastructure must be in order e.g. wifi

Q7 10 Make visible where you stand

Q7 11 You can sabotage innovation if you do not have this well organized. This must be clear to everyone 1.04.00

Q7 16 Involve people actively, but above all people must be active (entrepreneurship)

Q7 20 Vision of innovating is more important. Vision of learning to innovate distracts from the goal, delivering better care.

Q7 27 Board should give direction and facilitate 29.59 towards middle management and staff and communicate where are we going and ask to think with us

Q7 31 Not necessarily necessary

Q7 35 Taking time to engage with each other (easily overlooked). 21.21 Facilitating that is a good form to involve healthcare professionals

Q7 Innovation consultant. Being asked to organize peer review (accountability) 1 x month e.g. for project leaders when working differently in care.

Q7 Inspired by the pillars of innovation readiness (added is not ours) -> innovation maturity strategic direction/innovation organization/leadership/learning about and from innovating. Used to have the conversation among themselves during peer review.

Q7 Peter Hintzen: Day after tomorrow (book) too often 90% goes to the idea and 10% to the future. Shit of today - today - tomorrow - day after tomorrow.

Q7 With employees develop entrepreneurship -> more important almost than encouraging employees

Q8

Q8 27 Board/senior management need to get on board with it and how they encourage it is important. Innovation should not be non-committal and should be well invested in your organization and actually give space and not let it be too non-committal. And that role also includes encouraging employees, because that's where it should come from. If board and senior management does its job well, then you hope that middle management will take over/join this, that this will be arranged 28.10 to think and do.

Q8 24 There are people in every layer of the organization/specialty who get excited about innovating. 44.50 I don't really believe in a role you can play. I believe more in the right team composition and clear vision and direction. Then close people who belong to that and have a sense of it.45.20

Q8 What are we investing in and not investing in, does innovation match what is needed, does the innovation make impact etc.? There are many innovations, adoption rate is, entrepreneurs with good ideas are struggling to get through. Implement and scale up full potential innovations through money, knowledge of how to grow businesses, networking (8 vtt organization as a ring around it as implementation enhancers). Planning for a learning network. Shift from care to welfare is an interesting perspective.

Q8 1 Leadership matters. 33.35 director who states here is where we are going, and everything in the organization is linked to that. So you have to share with each other that mission, well with criticism that is allowed,

Q8 7 You have to do it as a team, it has to be in the culture. Good example symposium of VVT org E. There you saw the employees who were there, they had a certain DNA in them fed by the DNA of the director. Interdisciplinary. You want as many people as possible in the organization to have a landmark in an (innovation) team 51.12 e.g. on job level, or visibility, or credibility. The innovation team should have a good network in the organization to quickly assess and arrange things. They just know the organization. 52.20 That you can move quickly. That helps to retrieve information and develop proposals.

Q8 22 You have to do it as a team, it has to be in the culture. Good example symposium of VVT org E. There you saw the employees who were there, they had a certain DNA in them fed by the DNA of the director. Interdisciplinary everything needed.

Q8 4 Ability for people who really have this as their primary job, you need money for that.

Q8 2 Establish substantive innovation themes that make choices clear much more important than statement 3 describe what the organization means by innovation

Q8 18 Well important, however it is much more important that you are going to do something with it.

Q8 32 And the role of managers also includes stimulating employees, because that is where it has to come from.

Q8 10 Preconditions, you just have to have that should just be there. Think about the target groups in your organization is part of your innovation.

Q8 31 bv innovation lab that invites a different way of meeting than bv in the office and that it is somewhere else. That can be stimulating, well very normal in the process, actually it should just be there.

Q8 23 There are people in every layer of the organization / specialty who get excited about innovating. 44.50 I don't really believe in a role you can play. I believe more in the right team composition and clear vision and direction. Then close people who belong to that and make sense of it.45.20 QUOTE

Q8 30 Capturing learning experiences obviously important but courage is more important

Q8 28 There are people in every layer of the organization / specialty who get excited about innovating. 44.50 I don't really believe in a role you can play. I believe more in the right team composition and clear vision and direction. Then close people who belong to that and make sense of it.45.20

Q8 3 Vision and ambition are much more important

Q9

Q9 16 Actively involve employees from the shop floor in the innovation process: One of the pillars of its organization around innovation is the innovation climate on the shop floor. See also below.

Q9 12 Provide a toolbox of innovation tools: Too instrumental approach. Get employees on board first, and then that toolbox will come naturally.

Q9 28 Clear role for employees on innovation: One of the regional projects she leads is about the use of the caring IG expert innovation. That's a new position where a caring IG gets the opportunity to develop herself to an MBO certificate level on technology and innovation. That offers career prospects. So they are rostered out 4 hours a week to work on innovation. After all, you need someone where the work takes place who can actively engage in innovation. There is also an innovation team, but they are not part of the care sites, while the caregiver IG also just works in care. So she has those innovation glasses on throughout the work week and actually has 4 hours to contribute to all kinds of innovation projects at her own location. There are two innovation locations and per location there are two caring IG working with innovation. They are also investigating whether this has an effect on the willingness and ability to change (want, need, can); in what areas do they contribute to this.

Q9 1 Formulate an innovation ambition: Without a good ambition you will get nowhere. You have to have a dot on the horizon. A good vision around innovation is important, as far as she is concerned that is also part of this. And shaping your process. Consideration in decision-making regarding the choice of innovation projects. There must also be support within the board, that they propagate innovation.

Q9 19 Collaborate with external partners on innovation themes: Should also have been on 'most important', but there was no more room there. Contact with Health Holland and we also try to put the living labs away properly. We want to position ourselves more proactively, also towards the (Tech) companies. We want to communicate that the developments must go faster, instead of following the development agenda of the companies. Try out different Tech companies and see which one is the best, with which one you can connect well. We try to put that collaboration with external partners well away in living labs with education and research, with residents and loved ones themselves, with Tech companies. We are still learning how to get the most out of those living labs. It is not in our DNA to be proactive in that, so to put Tech companies to work instead of waiting to see what they come up with. Those are big steps and take effort.

Q9 10 Create a communication plan for innovation: Creating a good story both internally and externally is important. Have long been modest, but are now trying to get out better and earlier what they are doing. No 'making a shopping list', but 'presenting a delicious meal'. Internally, it still takes a lot of effort, including getting employees to think proactively. Chasing teams to post things e.g. on LinkedIn (which is sometimes better read than intranet). To make a good plan and line, we need communication much more. Has to put a lot of effort within her staff to get it done. So in that she now has to take some steps herself, which is why she put this under most important.

Q9 Learning and development is a stimulating factor, we do that well in our organization. But right now an impeding factor is the appreciation on the deployment of innovation. HR tools are not yet set up for innovation. No longer have performance and development interviews. Now periodically "the good conversation," but we haven't included the pillar of innovation in that yet. What does it do to you when we ask you to innovate? Is that a motivator or a stressor? Also deployed the dynamo questionnaire: one of the points that came out of that is that for employees, changes sometimes happen too fast.

Q9 31 Set up physical spaces in the organization for innovation activities: sees less value in this. Firmly believes that if you want to innovate, you have to organize support closer to the workplace. Where the work takes place (00:38:53).

Q9 18 Monitoring national innovation developments and trends: Not further discussed

Q9 3 Describe what the organization understands by innovation: Not further discussed

Q9 30 Record and evaluate learning experiences around innovation: Capture less relevant, that comes naturally with time.

Q9 Curious about the continuation of M's research and interested in the results of the scan that M is going to develop.

Q10

Q10 27 It starts with the top and management 17.32 vision formation if that is not there then everything stops 17.40 must create conditions and it must be clearly positioned in the organization and that you must create the climate for that 39.55 QUOTE

Q10 31 Least important but needed for trainings

Q10 18 care technology consultant, connecting link between technology and care teams. Actively looking for topics and opportunities on the market 43.05 cooperating colleague

Q10 Quality functioning since 2021 member of MT program manager processes, operations and care technology innovation in OZO. The biggest task in innovating is on the people side 31.00 and preconditions must be taken care of 3.15

Q10 20 Not yet concrete on how we want to innovate, but on care technology, as part of Waardigheid en Trots (Dignity and Proud) to get organization into a different trend of thinking. Awareness of innovation, enthusiasm, change aspects very important to get employees into a different mindset 19.49

Q10 OZO it is not so obvious to be busy with professional development (itt hospitals) and keep up. At OZO you come to do your thing and go home again (this strikes the respondent given his previous experiences) 21.15. Also as part of steering in history (little incentive on self-development) 22.30 and education level 2 and 3 factors at play 22.50 reflect contine process

Q10 28 RIZO it is not so obvious to be engaged in professional development (itt hospitals) and keep up. At RMP you come to do your thing and go home again (this strikes the respondent given his previous experiences) 21.15. Also sometimes as part of management in history (little incentive on self-development) 22.30 and education level 2 and 3 factors come into play 22.50. do they have this development need less? Focus on what it means for residents 27.00 a very strong incentive

Q10 And it needs to be clearly positioned

Q10 16 Focusing on what it means for residents 27.00 a very strong incentive. We are already 10 steps ahead as management and employees need to be well included in that. How to get a leadership group to do this and convey enthusiasm 41.15

Q10 26 Also like part of management in history (little incentive on self-development) 22.30 and education level 2 and 3 factors that come into play 22.50. do they have this development need less? Focus on what it means for residents 27.00 a very strong incentive

Q10 It starts at the top and management 17.32 vision formation if that's not there then everything stops 17.40 have to create conditions and it has to be clearly positioned in the organization and that you have to create the climate for that 39.55 and that it works well and that it fits well with what is happening in the rest of the organization. Innovation is not explicitly named.

Q10 30 Learning and experiments important though, documents no one is looking at, reflecting continuously is more important 45.30

Q10 29 Especially for management team, better if it is playful about department walk 47.50 have conversations 47.55

Q10 35 It is more part of, more important is learning attitude 47.01

Q10 15 Innovation dependent, when relevant then it becomes important, situational 46.40
