# Supplementary material for: Perspectives on managing innovation readiness in long-term care: a Q-methodology study
Source: BMC Geriatr. 2024 Dec 19;24:1017. doi: 10.1186/s12877-024-05572-3 (PMC11658053; doi:10.1186/s12877-024-05572-3)
Supplement: Supplementary file 3 — Additional file 3. [file 12877_2024_5572_MOESM3_ESM.docx]

Q11

Q11 16 Working on trust, safety, communication, and support. Support is one of the most important points. Let employees know they are experts, or that they represent a group (so consult with their constituency, take responsibility as an individual for your colleagues), and that the organization values their input (same as 25) 43.45. Also, let them know how support has already emerged in other parts/teams of the organization, for example, during a rollout. The role of ambassadors (can be one) and early adapters (who also provide feedback on how things are going) are important in this. 48.00.

Q11 15 Not that you skip them. But it's not always decisive.

Q11 8 Technical infrastructure can be very limiting for innovation, but it is not the most important thing.

Q11 34 Important. How can you make the next project better? Just as important as doing the project well. A good project is one that makes employees want to participate again next time. Innovating is a process.

Q11 25 Working on trust, communication, and support. Support is one of the most important points. Let employees know they are experts, or that they represent a group (so consult with their constituency, take responsibility as an individual for your colleagues), and that the organization values their input (same as 16).

Q11 26 To give backup and prioritize tasks within the team.

Q11 2 Clarify the question, why do I want to try something -> that determines whether something will be successful. Respondent/team receives 'assignments from the organization'. But their internal clients sometimes have already started, for example, tele-rehabilitation, without considering the why and the priorities. We bypass the objectives of the why.

Q11 35 To be able to take time, you need a budget. However, I find time more important. 35.50 So a pilot may have failed, but you can still have learned a lot. 1.00.00.

Q11 7 The strength must mainly come from the teams that want to innovate. Teams are needed to guide the organization, for example, from the staff. So setting up teams is important for support but not to replace the teams that have to or will do it themselves. 6.30.

Q11 33 The time for experimenting is over; it's more about trying out.

Q11 3 Definition of innovation, what is that, the respondent mentions that what this LRC organization is doing is not innovation, it's more about improving and renewing. How important is a definition actually? 50.17. It's mainly about the choices you make as an organization, same as 1.

Q11 31 Posters can be hung everywhere, space is not so important. The service is not space-bound. You do want to get out of the existing office setting. 51.30 for example, cushions in a corner. It can also be outside, it doesn't have to be a space.

Q11 28 This also applies to assigning tasks to employees. To be able to take time, you need a budget. However, I find time more important. 35.50, same as 6.

Q11 1 Definition of innovation, what is that, the respondent mentions that what this LRC organization is doing is not innovation, it's more about improving and renewing. How important is a definition actually? 50.17. It's mainly about the choices you make as an organization, same as 3.

Q11 13 From the top, yes, from the bottom, no. Because the professionalization step needs to be taken first at the bottom. Identify the biggest gains at the bottom. Be careful that an innovation process does not become an evaluation process; be careful with that, it can be sensitive at the bottom.

Q11 18 We are not there yet in LTC. It's important that you can, but not to become IR. In the region with LTC, we don't yet dare to trust each other regarding innovation (provides an example of collaboration on introducing ECD). Everyone wants to go their own way.

Q11 21 You want people who stimulate employees to come up with innovative ideas. And you need knowledge for that 12.20. You don't just benefit from stimulating ideas. A pile of ideas alone is useless.

Q11 4 To be able to take time, you need a budget. However, I find time more important. 35.50.

Q11 6 This also applies to assigning tasks to employees. To be able to take time, you need a budget. However, I find time more important. 35.50, same as 28.

Q12

Q12 22 Compose interdisciplinary innovation teams: believes this factor is related to the factor 'Actively involving employees from the shop floor.' Because if you put together an interdisciplinary team, you do it with employees from the shop floor. You do it together with the care professionals. These two factors are the same for him. If employees have come up with something themselves, the implementation goes better.

Q12 15 Involving family and loved ones in innovation: Takes organizational context into account in the assessment. Explains that the care organization works on an outpatient basis. Involving clients and loved ones in innovation is not realistic with 6000 clients. And in their small clinic (for people with dementia with behavioral problems), there is also little (mental) space for this. These people are all in crisis.

Q12 Not enthusiastic about the term innovation 'unit'.

Q12 Talking about 'time allocation' finds it a dead end.

Q12 Factor 20: 'A vision on learning': meh, there are so many learning methods.

Q12 Factor 28: A 'clear role': no, keep it nice and vague. Whoever wants to join, joins.

Q12 Factor 3: 'Describing what the organization means by innovation': that's 'stubborn' and forget it.

Q12 Factor 31: 'Physical space' would help, but I don't have it.

Q12 Factor 29: 'Reflecting on the processes in the organization': sees it happening more and more.

Q12 Considers 'consulting with employees from the shop floor' and 'composing interdisciplinary innovation teams' to be more or less the same.

Q12 Reflects on the nature of learning processes: learning is important, but people in LTC have a work-learning attitude: learning in the workplace, not too much via education.

Q12 Two factors: it's about the business model and market approach. They need to learn that they are responsible for the business they are running.

Q12 Composing interdisciplinary innovation teams: believes this factor is related to the factor 'Actively involving employees from the shop floor.' Because if you put together an interdisciplinary team, you do it with employees from the shop floor. You do it together with the care professionals. These two factors are the same for him. If employees have come up with something themselves, the implementation goes better.

Q12 16 Subsidies for research. Is that possible? Or are you allowed to do that? Shouldn't you find out first? I would prefer to see the subsidy process translated into innovation (in the elderly care organization), translated to the healthcare organization. But subsidies come from different pockets. It comes from different pots. And we, as an organization, don't understand that well.

Q12 14 A joint responsibility: explains that the first innovation plan is related to individual behavior and the motivation to want to innovate. You need employees who want to do it. You need to let them innovate. Give them that space. 18.00.

Q12 1 Composing interdisciplinary innovation teams: believes this factor is related to the factor 'Actively involving employees from the shop floor.' Because if you put together an interdisciplinary team, you do it with employees from the shop floor. You do it together with the care professionals. These two factors are the same for him. If employees have come up with something themselves, the implementation goes better. Same as 22.

Q12 18 Every employee should be given the opportunity to innovate, given the time for it.

Q12 Two factors: it's about the business model and market approach. They need to learn that they are responsible for the business they are running.

Q12 Not enthusiastic about the term innovation 'unit'.

Q12 Factor 28: A 'clear role': no, keep it nice and vague. Whoever wants to join, joins.

Q12 20 Subsidies for research. Is that possible? Or are you allowed to do that? Shouldn't you find out first? I would prefer to see the subsidy process translated into innovation (in the elderly care organization), translated to the healthcare organization. But subsidies come from different pockets. It comes from different pots. And we, as an organization, don't understand that well. Same as 16.

Q12 Factor 3: 'Describing what the organization means by innovation': that's 'stubborn' and forget it.

Q12 5 A joint responsibility: explains that the first innovation plan is related to individual behavior and the motivation to want to innovate. You need employees who want to do it. You need to let them innovate. Give them that space. 18.00. Same as 14.

Q12 Talking about 'time allocation' finds it a dead end.

Q12 Factor 31: 'Physical space' would help, but I don't have it.

Q12 Factor 29: 'Reflecting on the processes in the organization': sees it happening more and more.

Q12 7 Involving family and loved ones in innovation: Takes organizational context into account in the assessment. Explains that the care organization works on an outpatient basis. Involving clients and loved ones in innovation is not realistic with 6000 clients. And in their small clinic (for people with dementia with behavioral problems), there is also little (mental) space for this. These people are all in crisis. Same as 15.

Q12 A clear role: no, keep it nice and vague. Whoever wants to join, joins.

Q12 Factor 20: 'A vision on learning': meh, there are so many learning methods.

Q12 Talking about 'time allocation' finds it a dead end.

Q12 28 Reflects on the nature of learning processes: learning is important, but people in LTC have a work-learning attitude: learning in the workplace, not too much via education.

Q12 Not enthusiastic about the term innovation 'unit'.

Q13

Q13 34 There's a level of vulnerability in innovation; you have to be willing to look at what's not working well, a fundamental requirement at the organizational level. When implementing innovation, you don't necessarily know if it will have an effect. It's a means aimed at a goal.

Q13 5 What does a plan ensuring this not say? Innovating is dynamic; in a plan, you commit to what you've already scheduled. You can commit to doing it, but not necessarily how and when, which is also true in healthcare. You have no idea what, for example, a care robot will mean for nursing deployment.

Q13 Innovation is an ongoing task for the organization, renewing to fulfill its core mission. Employees are trained with a focus on tasks; should they be trained more mission-oriented instead?

Q13 15 Innovation is done primarily to maintain good, valuable, and affordable care for people.

Q13 16 Innovation comes from the participants by informing and inspiring; they implement it and deal with execution challenges. Middle management are also employees; should you divide that? Why assign certain tasks to middle management, an outdated way of thinking? Similar to 24.

Q13 35 When implementing innovation, you don't necessarily know if it will have an effect. Gain experience.

Q13 21 Equipping employees to innovate (how and preparing as individuals) on how you can act in your own context. Employees are trained with a focus on tasks; should they be trained more mission-oriented instead?

Q13 11 Determining together how you're going to do things.

Q13 7 Similar argumentation as 4.

Q13 When you budget separately, it becomes something distinct; separate activities that you then measure. However, you want to achieve that employees who feel called to do so have the space instead of having to use the innovation fund for that purpose.

Q13 23 Middle management are also employees and stakeholders in the organization.

Q13 24 Innovation comes from the participants by informing and inspiring; they implement it and deal with execution challenges. Middle management are also employees; should you divide that? Why assign certain tasks to middle management, an outdated way of thinking? Similar to 16.

Q13 26 Similar argumentation as 4.

Q13 When you budget separately, it becomes something distinct; separate activities that you then measure. However, you want to achieve that employees who feel called to do so have the space instead of having to use the innovation fund for that purpose.

Q13 4 When you budget separately, it becomes something distinct; separate activities that you then measure. However, you want to achieve that employees who feel called to do so have the space instead of having to use the innovation fund for that purpose.

Q14

Q14 27 Management emphasizes that innovation is a priority for the organization: Our director fully supports the work we do in research, development, and innovation. That helps a lot. Elderly care is still quite traditional and hierarchical. But if she emphasizes this, much more can happen immediately. Then people are also more motivated to do it. Care workers are often stubborn and passionate about their profession. They don't like to be restricted by rules. You have to make clever use of this passion. From management, you should also give employees enough space for this. Not just top-down management, which doesn't work. Different management policies are needed: they are currently judged a lot on practical matters but not evaluated on innovation.

Q14 12 Provide a toolbox of innovation tools: a toolbox is limiting. "Here's one for that purpose, see what's inside and good luck." Developing a good toolbox is very difficult and can be too limiting for people.

Q14 26 Middle management creates an attractive innovation climate for employees: A lot depends on middle management. Whether initiatives can succeed or not often depends on them. Sometimes they fail to make an initiative thrive. Sometimes they want to, but they are also judged on completing staffing requirements. Not too much vacancy, not too much absenteeism, etc. But they are often not evaluated on innovation. Therefore, they prioritize other matters when it comes down to it, which is understandable.

Q14

Q14 More generally (not necessarily just middle management): Within the organization, priority is often given to short-term client care rather than innovation, which is beneficial in the long run. (18:46)

Q14 16 Actively involve frontline employees in the innovation process: Employees must be involved, preferably from the beginning, to be motivated to participate in such things. It also works a lot from the bottom up: what do employees like, what challenges do they face, etc.

Q14

Q14 Set up a learning point at one of the locations where students/interns receive practical research lessons on Monday afternoons. They often also carry out a small research project focused on innovation. For example, why is this innovation tool unused in the closet? So very practical and close to the work floor, so other employees are also included in innovative thinking. And policy/management also gains insight into what is happening and why things are not being used. This idea also came from the employees themselves, to set it up like this.

Q14

Q14 Factor 27, 26, and 16 together: In the interaction between management, middle management, and employees, it may or may not succeed. Preconditions such as money are also necessary, but if people aren't motivated to do something, nothing will happen. Organize an annual master class on innovation, including critical and innovative thinking. Everyone in the organization can sign up for this, so that 'innovation' spreads like wildfire throughout the organization.

Q14 27 Factor 27, 26, and 16 together: In the interaction between management, middle management, and employees, it may or may not succeed. Preconditions such as money are also necessary, but if people aren't motivated to do something, nothing will happen. Organize an annual master class on innovation, including critical and innovative thinking. Everyone in the organization can sign up for this, so that 'innovation' spreads like wildfire throughout the organization.

Q14 26 Factor 27, 26, and 16 together: In the interaction between management, middle management, and employees, it may or may not succeed. Preconditions such as money are also necessary, but if people aren't motivated to do something, nothing will happen. Organize an annual master class on innovation, including critical and innovative thinking. Everyone in the organization can sign up for this, so that 'innovation' spreads like wildfire throughout the organization.

Q14 4 Budget: Sometimes a stumbling block, but they have someone on staff who is great at applying for subsidies, so they can always free up money for innovation. It often involves subsidies provided by the healthcare office or the municipality. A colleague knows her way around this very well, where she can apply for subsidies. Strategy determines what budget to look for and also what concerns employees. If they see the added value, they are more motivated to cooperate.

Q14 32 Encourage employees to take initiative with innovation themselves: see also factor 16.

Q14 17 Factor 19 and 17: She says they also share knowledge within their SANO network via linking pins. They work closely with other healthcare organizations, external partners, Leiden University, The Hague University of Applied Sciences, and the ROC in The Hague. ROC is involved in research and innovation at the MBO level.

Q14 19 Factor 19 and 17: She says they also share knowledge within their SANO network via linking pins. They work closely with other healthcare organizations, external partners, Leiden University, The Hague University of Applied Sciences, and the ROC in The Hague. ROC is involved in research and innovation at the MBO level.

Q14 30 Factor 30 and 34: Learning from the process itself is also important, even if it 'fails'. Not everyone has that mindset yet. Elderly care may need to be structured differently, also at the industry and government levels, so that frameworks are set in a way that allows people to approach things innovatively. Right now, it's too limiting.

Q14 34 Factor 30 and 34: Learning from the process itself is also important, even if it 'fails'. Not everyone has that mindset yet. Elderly care may need to be structured differently, also at the industry and government levels, so that frameworks are set in a way that allows people to approach things innovatively. Right now, it's too limiting.

Q14 They do organize an annual master class on innovation, including critical and innovative thinking. Everyone in the organization can sign up for this. Because you want it to spread throughout the organization like an oil slick. This master class is conducted by an external agency, in collaboration with other healthcare organizations and with municipal subsidy.

Q14 13 Establish an innovation process: it's not limited to a specific process. That's another 'framework'.

Q14 31 Physical spaces: old-fashioned, but that's a 'generation thing'. Older colleagues prefer a 'brainstorm space', while younger people (and they themselves) don't see the added value as much. They value more flexibility with telecommuting/flex spaces, etc. Those groups that appreciate such a space are often not the ones with the most innovative ideas.

Q14 8 Technical infrastructure: less important because innovation is often not technical.

Q14 24 Factor 24: Clear role of middle management less important, because you have to consider it individually. There are also managers who are not interested in that and are not suitable for it.

Q14 5 A multi-year plan: then you tie it down too much.

Q14 3 Factor 3: "Defining what the organization understands as innovation" is not considered very important by her because it can restrict people. She feels that employees on the shop floor don't understand it. The translation towards employees is not done well. It can stifle free thinking and creativity of people. She also mentions that their own organizational strategy didn't sit well with the employees because they felt they were already doing everything in it. Don't make it too complicated for the employees. The ideas that people come up with already fit within the strategy, so why impose such a framework on them?

Q15

Q15 5 A multi-year plan: It starts with making a good multi-year plan and vision for innovation supported by board and management.

Q15 31 Setting up physical spaces: He does not believe that you really need to have a physical space where you try out innovations etc. You can do that in daily practice as well.

Q15 13 Setting up innovation process: The second thing that follows from this (from the plan and vision) is the process: how do you deploy innovations now. What do we think of the innovation? With a positive result, you make a good implementation plan, to which you link a project team.

Q15 When you want to continue with an innovation after a positive result, you have to have the ability to set up a project team that will do the work. This is where things often go wrong. In fact, you should place this as little as possible with employees/officials who "already have a job. If you have to do it in addition to your daily work, it regularly goes wrong. See also factor 4.

Q15 1 Formulate innovation ambition: See also factor 5

Q15 11 Agreements decision-making in innovation process: See also factor 13

Q15 4 Making budget available: There must be enough capacity in formation and in money. Innovation is often assigned to a middle manager who already has a busy schedule. He then has to do this within his time with his own staff and resources, and that is prohibitive.

Q15 17 Factor 17 and 19: Networking and learning from each other is also important, so that you don't all start researching the same thing. However, in this you often have to deal with the wilfulness of directors, who still want to "score" with something. After a change of directors, there is often a shift from cooperation and networking to 'pulling up the walls'. Having trust in each other is a big factor here and sharing information back and forth. And should you set up that collaboration formally or informally? Has both advantages and disadvantages. A formal form of cooperation creates more commitment. In South Limburg we are less good at cooperating and consulting with each other. The competition between organizations also plays a role in this, for example in the decision to all choose a different EPD system. There is a complex healthcare landscape in Limburg.

Q15 19 Factor 17 and 19: Networking and learning from each other is also important, so that you don't all investigate the same thing. Here, however, you often have to deal with the wilfulness of directors, who still want to 'score' with something. After a change of directors, there is often a shift from cooperation and networking to 'pulling up the walls'. Having trust in each other is a big factor here and sharing information back and forth. And should you set up that collaboration formally or informally? Has both advantages and disadvantages. A formal form of cooperation creates more commitment. In South Limburg we are less good at cooperating and consulting with each other. The competition between organizations also plays a role in this, for example in the decision to all choose a different EPD system. There is a complex healthcare landscape in Limburg.

Q15 7 Factor 7: setting up innovation teams. He wonders if this is the right way to go. Depends on how you approach it. He himself has had less good experiences with teams composed as a broad reflection of the organization. That doesn't work so well and doesn't deliver the things you expect. Look for more specialized people working on innovation, but from different parts of the organization. Truly reflecting the organization makes little sense. Rather look for certain themes and put people together who know something about them.

Q15 33 Factor 33: Having guts (score 0, neutral) is very much related to 'how are you covered'? How does management stand on it? Are things allowed to go wrong? Is room given to experiment? Isn't everything beaten to death with business cases to follow? As soon as that threshold is removed, you can be more daring. Business cases often look at the money, while there are other factors by which you can measure the success of an innovation. Many people get involved. The person who has innovation in the portfolio has to have a lot of regulatory power to be able to have a certain freedom, linked to a budget.

Q15 36 Factor 36: Learning (score 0, neutral) is certainly also important, but you don't necessarily have to set up whole tools for that. If you have a permanent team of people who are freed up for innovation and who can do this for a few years, then you automatically secure the knowledge.

Q15 16 Actively involve employees on the shop floor: He has little confidence in the input employees and loved ones can give regarding innovations. Unless you frame it very much, pretty little comes out of that. In his experience, they find it difficult to think along. You already have to frame it per theme, but on an "open question" regarding innovations, you have to give them an answer.

Q15 15 Involve family and relatives: See factor 16

Q15 12 Make toolbox available: Not as necessary.

Q15 29 Reflecting on innovation maturity: What's in it for you? Emphasized. It's not a priority to have a reflection on that. You actually do that throughout the process, therefore it's not something very special. (27:27)

Q15 Factor 23: Regarding middle management (score -2): he does not have much confidence in the innovativeness of this group. It also depends on the opportunities offered to be innovative (27:53).
