# Supplementary material for: Perspectives on managing innovation readiness in long-term care: a Q-methodology study
Source: BMC Geriatr. 2024 Dec 19;24:1017. doi: 10.1186/s12877-024-05572-3 (PMC11658053; doi:10.1186/s12877-024-05572-3)
Supplement: Supplementary file 4 — Additional file 4. [file 12877_2024_5572_MOESM4_ESM.docx]

Q16

Q16 1 This is where it starts: innovation starts with ambition, the vision for it, what do we want, what is its importance

Q16 31 Space can facilitate, but not the most important thing

Q16 Ranking factors: in training works a lot with designers, to learn in an innovation process, iterative process very important.

Q16 4 You have to allocate time resources money and resources if you are serious in innovating, you have to facilitate that 29.20 with this you indicate how serious you are in it

Q16 16 All 3 of these things are about employees at 3. How do you organize that: involve employees, not necessarily that they are responsible, but they are the ones who do the primary process and thus produce the result?

Q16 6 All 3 of these things are about employees on 3. How do you organize that then: involve employees, not necessarily that they are responsible, but are the ones who do the primary process and thus put down the result

Q16 25 All 3 of these things are about employees on 3. How do you organize that then: involve employees, not necessarily that they are responsible, but are the ones who do the primary process and thus put down the result

Q16 24 In row 2 The responsibility to set up an innovation process lies more with the management, that doesn't have to be asked of everyone. The knowledge provided by the organization is also necessary for this. Innovation belongs to the whole organization

Q16 23 In row 2 The responsibility for setting up an innovation process lies more with management, although this need not be demanded of everyone. Providing the knowledge by the organization is also necessary for that. Innovation belongs to the whole organization

Q16 27 In row 2 The responsibility for setting up an innovation process lies more with management, although this need not be demanded of everyone. Providing the knowledge by the organization is also necessary for this. Innovation belongs to the whole organization. Board & senior mng, the support really has to be there as a condition that supports, that radiates that you really care. So not just go and play and see what comes out of it.

Q16 36 In row 1 focused on learning

Q16 20 In row 1 focused on learning

Q16 2 On making innovation policy concrete for people 41.30

Q16 14 You will talk about that often

Q16 18 Nice to map, you have to start with your own ambition

Q16 8 Can but not all-important

Q16 21 Education depending on what is needed. Education relevant though

Q16 10 More internal, what is it what are we going to do, where can you find it

Q16 13 No belief in whole 'blue' approach, learning climate and experimentation space is more important

Q16 34 Interesting: what can you learn from 'failed' innovation, great success if you can learn from it

Q17

Q17 1 Factor 1: Formulating innovation ambition provides clarity: what is our problem and how are we going to address it?

Q17 2 Establish content themes: Setting content themes is important, otherwise you won't know where to focus. Where are the problems and how can innovation help us address them? (30:50)

Q17 4 Factor 4: Budget is very important: now a lot leans on subsidies, but not enough thought is given to: what if the subsidy stops? Then there has to be budget.

Q17 5 Factor 5: Multi-year plan: innovation is a rather abstract process that cannot always be molded into a multi-year plan (45:08). Can also inhibit, because you stick too much to the plan.

Q17 6 Agreements about tasks and position of employees: Making agreements about the position and tasks of employees is important, because if you don't, innovation gets bogged down in their work. People must be given clear time and space to work on it. If people are not given hours (for a certain period of time), they cannot make strides either (39:09).

Q17 7 Factor 7: Innovation teams: consist not only of professionals, but also of clients and loved ones. As broad as possible, also within the chain (hospitals etc.). Setting up innovation teams is less important. You have to see per organization what the culture is and what strategy fits best.

Q17 8 Factor 8: Getting technical infrastructure in order is important, often the wifi within the organization is very poor. Then you are already 10-0 behind.

Q17 9 Make innovation knowledge available: Less important, not explained in detail.

Q17 10 Factor 10: Communication strategy you have to make and then from this comes a communication plan.

Q17 12 Factor 12: Toolbox is important, to help with the steps to take and where to start.

Q17 13 Setting up innovation process: Making agreements about decision-making and setting up innovation process is important, because otherwise pilots and projects run too long. There is not always (timely) evaluation. You have to constantly evaluate innovation and implementation to learn from it.

Q17 15 Factor 15: You have to involve family and relatives as well, because this group in particular can become resistant if they do not understand why an innovation is needed (23:48).

Q17 16 Factor 16: Involve employees in innovation process: they need to know that there is a process and that they are allowed to sign up for it and contribute ideas. But this is not necessary for all employees. Do come into contact with it afterwards.

Q17 18 Monitoring developments and trends: Less important, not explained in detail.

Q17 19 Factor 19: Collaboration is important, but less so when you start innovating.

Q17 21 Education: is especially important for the "innovation people. So more important for a project leader than for all employees.

Q17 23 Factor 26 (and 23): Middle managers need to ensure attractive innovation climate is important, because if they don't, nothing happens. Employees are sucked into the delusion of the day. How people are trained also plays an important role in this. How innovative are you in finding solutions to issues?

Q17 25 Factor 25: Valuing employees is very important: that's how you get them on board and start to grow.

Q17 26 Factor 26 (and 23): Middle managers need to provide attractive innovation climate is important, because if they don't, nothing happens. Employees are sucked into the delusion of the day. How people are trained also plays an important role in this. How innovative are you in finding solutions to issues?

Q17 27 Board carries out innovate priority: this is where it starts. Defining what organization understands by innovate is important, because there must be a clear vision. This must be supported within board, otherwise the organization will not move forward. If management does not make it clear that innovation is a priority, you will suffer later in the process. Middle management is also important here. They often have to 'add' innovation processes to their daily routine. They are sometimes not sufficiently facilitated in this process. Choices can sometimes have short-term consequences for the financial returns (the amount of production turned over), but have long-term advantages. If this is supported by the board, it is easier for managers to make choices. QUOTE

Q17 31 Furnish physical spaces: physical spaces are somewhat less important. The best is when people can try out in practice.

Q17 32 Encouraging employees to work on their own: She thinks the vehicle is employees the best. She believes that if you include employees well and give them space and facilitate them that they can be the flywheel for an innovation. And not the organization itself. It starts with employees, that's what it's all about. In addition to having an ambition and vision, it is the employees who ultimately have to do it.

Q17 33 Factor 33: Having guts: you just have to do it. This is something that is sometimes lacking. Not because they don't want to, but from a financial point of view. If an organization is running badly, then innovating is the first thing to be stopped (34:04).

Q17 34 Learning from mistakes made: Learning from mistakes made is only done at a later stage.

Q17 35 Factor 35: Taking time to learn is important; you need to be facilitated to engage in it.

Q17 36 Factor 36: Learning from each other is important, because now everyone is still on their own little island 34:31). Sometimes everyone in the region is doing the same thing.

Q18

qsort2 statements2 explanation

Q18 3 Defining what organization understands by innovation: first of all you have to know what innovation is, what you understand by it.

Q18 34 Learning from mistakes made: thinks learning is also an important factor, but you do this as you go along in the process.

Q18 She is trying to reason out what the logical steps are in the process to be taken. Sits with this for a while and often changes how she puts the cards down. She works from the preconditions to the content, she notices herself. Because normally she always starts from the content. But she thinks that to be 'ready' to get started with activities, you first have to have the preconditions in order. So these are usually listed under 'most important'. From that then follows what you need to start doing, what you need to have available, etc. So she ranked them in the order in which you need to do things to become innovation ready. So the cards describe from right to left the timeline you have to follow, so to speak.

Q18 From score 2 she names this as: everyone knows what to do, there is money (because without a budget nothing happens), you agree with each other what you want to work towards, you start coloring outside the lines (guts). What exactly are we going to do? People want substantive guidance, so you're going to establish that. And you're going to do that in a learning way. Agree who is going to be in charge of what and what exactly you are going to do. Meanwhile, keep an eye on national developments and know how you relate to them. Assemble teams (often interdisciplinary) and communicate about it. Set up education for it. See if you can use certain tools/toolbox, so that the teams can work hands on. Also learn from other partners and seek collaboration. Chart progress, reflect on it, learn from it and record it. Central management learns to create an attractive climate for innovation, so that more people want to participate. Based on what you have learned, you make a long-term plan. You put your technical infrastructure in order and possibly create a place where you can come together. And finally, you share what you've learned with others.

Q18 1 Formulate innovation ambition: You need to know: what do we want to do, why are we going to do that, why do we want that? See also factor 27.

Q18 16 Actively involve employees in process: Everyone has something to do in it, everyone gets a role. First you have to involve employees from all layers in the bigger story and in the ambition, and then you start specifying what everyone has to do (clear role on innovation, score 2).

Q18 15 Involve family and loved ones: Everyone has something to do in it, so family and loved ones also get a role. You have to see the participation council and client council as your best ally, because they also want to think about how things can be done differently and better.

Q18 27 Board conveys that innovation is priority: Convey: we all think it's important. In the organization where she herself works, there was a lack of managerial commitment. They worked in a project-oriented way, but employees had no idea whether what they were doing was valuable and within which frameworks they could operate. To get something off the ground, you do need this foothold. Organization must frame innovation positively and not hide behind rules and quality standards etc., and that task also lies with the board.

Q18 33 Factor 33: What do we think, what do we stand for? Can we argue that and address it in a safe, responsible way anyway? You have to stand up to rules and laws, but not hide behind them. The organizations that are moving forward don't either, but the organizations that are standing still say, 'no time, no money, it's not allowed, it can't be done.'

Q18 9 Make innovation knowledge available: Again, you do this as you go along in the process, when you yourself have already taken steps in the process and learned things from it.

Q18 8 Technical infrastructure: Especially important with technical innovations. So depends on what you are going to do.

Q18 31 Setting up physical spaces: You don't always need those.

Q18 5 Create multi-year plan for innovation: That will come later in the process.

Q19

Q19 27 Board conveys that innovation is a priority: you need a clear strategy as an organization. Board needs to point the organization's compass in the right direction. How can I make my healthcare organization more future-proof? QUOTE

Q19 31 Physical spaces: About this and the 4 factors below that he placed most to the left, he says that these factors are not LESS important, but OTHER important. These only come into play in the 2nd or 3rd phase of innovation.

Q19 - System innovation, with which you look at the system world through a different lens (e.g. agree on a different way of funding with insurers).

Q19 - Social innovation: using scarce human resources in a smarter way.

Q19 - Technological innovation: using tools

Q19 - Cultural innovation: trying to change something in the DNA/mindset of your organization

Q19 The most successful innovations have all these four elements in them and are interrelated.

Q19 Example from our own organization is to stop working with 'hourly billing' within district nursing. Other agreements have been made with the insurer. They are now no longer paid by the hour, but by the month (system innovation). This way of working means that the more we unburden (don't 'pamper' too much), the better our earnings model is. This is another cultural innovation, different way of thinking. It means that some things like improving well-being etc. do not necessarily have to be done by care people. This gives another view of the labor market and also of the possibilities of using technology.

Q19 These kinds of innovations where everything is connected create the biggest changes. According to him, innovation is mainly about creating movement, including by having the right conversations with each other.

Q19 To this end, engage in the conversation with others, among other things, to gain confidence, a critical eye and ensure that innovation becomes everyone's business.

Q19 33 Have guts: If you are convinced of something, just go do it. If it doesn't work: you also learn from brilliant failures. It did help you figure out what does work. So it does help you move forward. That is also part of innovation: allowing yourself to fail. And managing expectations. Sometimes you just don't know yet whether something will work, but you need the 'Pippi Longstocking attitude'. And allow yourself a living lab, where you can experiment without a failure having too big an impact on the organization.

Q19 28 Clear role of employees: An innovation must land in the capillaries of your organization, because that is where the difference is made. Innovation should be something of people and not of systems.

Q19 It is important to involve the participation council as early as possible, so that they feel that the innovation belongs to them as well. Include them in ideation and policy development. This way you increase support and the chance of success of innovation. And sometimes you get useful advice and ideas. You need 'ordinary' people who look at innovations through 'ordinary' glasses in order to think about them. And who also dare to be critical. If you can get these people on board, it increases the chances of success. If you don't, the chance of success is undermined.

Q19 23 Provide and support middle management with knowledge: you want to help them solve problems in the first place. And you are never against a solution that you have come up with yourself. Have a conversation with people: what are you running into? Irritation leads to innovation. So go looking for things from people's lifeworld and see if you can get the system world to come up with a solution for the problems from the lifeworld. quote

Q19 26 Middle management ensures attractive innovation climate: Managers are crucial in this, because they have to have the right conversation about this with their employees. Within the range of the direction of the compass (which the board determines), people are needed who translate this direction into a roadmap.

Q19 4 Factor 4: Whether there is a budget for an innovation does not interest him that much. If you let innovation depend on having/not having a budget, then you are obviously not intrinsically motivated. Your innovations should be derived from your beliefs that you believe in and stand for. How can I make my healthcare organization more future-proof? Once I know that and have a solution for it, I'll look for funding after that. And not the other way around. Subsidy programs therefore often don't work in his eyes.

Q19 19 Cooperation with external partners: See factor 31

Q19 18 Monitoring national developments: See factor 31

Q19 17 Exchange innovation knowledge: See factor 31

Q19 15 Involve family and relatives: See factor 31

Q20

Q20 22 Yes very important to learn from each other and you have to do that together

Q20 31 You have to facilitate that what the organization needs, can also be in the park

Q20 13 A bit blue you have to arrange it however you have to give employees especially the possibility to let them realize their own innovation process.8.04

Q20 Grants you have to have a lot of knowledge for that to be able to apply for that 46.10 bvzonmw takes a lot of time and whether you get it and if you get it then the leave hours are not funded. 46.53

Q20 Innovation is doing something the other person hasn't done yet

Q20 Innovation is something you have to do and learn

Q20 The professional must become enthusiastic about it

Q20 16 Employees must be able to put their question, have dialogue around innovations 17.41 respondent thinks it is important that employees are good in their role 36.50

Q20 20 Learning is important also think about reflection 37.07 learning from mistakes made. This organization is especially concerned with how to learn from adoption/adaptation of innovations37.58 from the perspective of employees. How do you get employees to use and accept an innovation as part of their work 38.11 what are the success and failure factors 38.43 organizing iterative reflection process also applies to innovation implementation 39.20

Q20 19 Learning from each other, especially if you want to innovate faster, that one does A and other implement B and learn from that

Q20 33 That space must be there

Q20 29 Reflection precedes learning from it16.50

Q20 35 Opportunity and motivation is important 14.22 just like competencies, taking time is evident in elder care 14.30 and that is a big problem

Q20 25 Yes employees need to be hoisted on the soapbox for the efforts they make in terms of innovating

Q20 5 From the demand that the organization has the beckoning perspective interpret 18.50

Q20 27 Important not all-important, Employees must be receptive to it

Q20 32 As employees become more curious and a culture of how can we solve this this helps with adaptation of innovation. Let them experiment and get started 15.21

Q20 24 Not cast in stone role is innovation dependent 9.30

Q20 36 Yes learning from each other very important

Q20 23 It doesn't necessarily have to be through management, can also be through matrix structure and disciplinary teams. Maybe the manager is not enthusiastic at all. Enthusiasm is very important.

Q20 14 Overview and insight yes if this leads to a blue control mechanism then respondent thinks this is too limited. It must create frameworks, sometimes things fly out of the corner, you must be able to create space 20.00

Q20 18 Yes important, but beware me too, you have to have a reason as an organization

Q20 8 If technical had been in brackets this factor would have been on more important

Q20 6 You have to give employees room to experiment, to walk outside the path, you don't want to fix that 42.15

Q20 7 Never separate in the organization, mixing team with experienced and less experienced people in terms of innovating to learn

Q20 10 Something blue, better yet communication strategy

Q20 21 Facilitating element of curiosity about innovating and that learning

Q20 15 Depends on type of innovation, if they notice something about it yes

Q20 12 Yes but not as doch ma

Q20 2 Yes but always in relation to the direction of the organization

Q20 11 Yes you must do, prevent me from making a 1000 flowers bloom
