# Supplementary material for: Perspectives on managing innovation readiness in long-term care: a Q-methodology study
Source: BMC Geriatr. 2024 Dec 19;24:1017. doi: 10.1186/s12877-024-05572-3 (PMC11658053; doi:10.1186/s12877-024-05572-3)
Supplement: Supplementary file 5 — Additional file 5. [file 12877_2024_5572_MOESM5_ESM.docx]

Q21

Q21 1 Factor 1 Innovation ambition and Factor 2 content innovation themes: sits more at board and management level. Also important. You also need vision and commitment from the board. You establish the themes by listening carefully to your employees and what they need and so give them input into this. As a board, you always have to build that bridge towards the employees.

Q21 3 Describing what the organization understands by innovation: I don't have much of a problem with that. You can describe this in all sorts of documents, but what use is that to the employee on the floor?

Q21 4 Factor 4 Budget: is important, but it is mainly about freeing up time. But an innovation should not cost too much time and space.

Q21 5 Long-term plan: Don't really believe in it. It is good to have a vision and to stimulate innovation, but you don't have to specify this in a multi-year plan.

Q21 7 Setting up innovation teams: you should not innovate on 'an island'. You have to do it together with all stakeholders who have to use the innovation. Thinking it out or trying it out by a select club can be smart in some cases, but if you pull it apart too much you create a gap that you have to start filling again. It is therefore better to involve everyone who is willing and able in innovations as much and as early as possible. Then you also know immediately whether it works or not.

Q21 8 Factor 8: technical infrastructure should really be in order for certain types of innovations.

Q21 9 Factor 9: again has to do with the learning process.

Q21 10 Factor 10: should speak for itself that innovation is important. Communication plan should not be necessary.

Q21 11 Decision-making arrangements: Also depends on the other factors mentioned here. Who has what role in decision-making and where are these decisions made? You should also organize this lower in the organization, so also give employees roles regarding decision-making. Or at least involve them for relevant input. Also from the idea that the innovation must be useful for the employees themselves and they have to work with it. If you do not involve employees in the decision-making process, you may make the wrong assumptions and estimates. Both in technology 'push' innovations (the introduction of new care technology, technology is central to this) and in the more process-oriented innovations ('pull'; what problem are we facing and how are we going to solve it) you have to involve employees. He himself believes more in the latter form: innovate from set goals rather than from technology.

Q21 12 Toolbox: Can be relevant, but also tends toward 'this works elsewhere, so I'm going to try to implement it exactly like this in my organization.' He doesn't believe in that. You first have to be very clear what you yourself need, and then you can go and get inspiration from others and from toolboxes. But it is not a matter of 'copy paste'. Although, of course, you don't always have to reinvent the wheel. But a toolbox is not blissful.

Q21 13 Factor 13, 14, 30, 34, 36 Learning: also important. Share experiences, evaluate, reflect. Provides incentive to stay motivated in the face of setbacks. Innovation is an iterative process: constantly adjust and improve your process (factor 13). This process can be slightly different for each project. It should be servant-driven and therefore not rigid. Keeping an overview (factor 14) is what middle management and the healthcare professional do, and they offer this as input to the board.

Q21 14 Factor 13, 14, 30, 34, 36 Learning: also important. Share experiences, evaluate, reflect. Provides incentive to stay motivated in the face of adversity. Innovation is an iterative process: constantly adjust and improve your process (factor 13). This process can be slightly different for each project. It should be servant-driven and therefore not rigid. Keeping an overview (factor 14) is what middle management and the healthcare professional do and they offer this as input to the board.

Q21 17 Share innovation knowledge: For example, sharing knowledge gained with Vilans. Do you benefit from that yourself? The other way around, perhaps; using Vilans' knowledge. So it's not not useful, but you shouldn't expect too much of it either.

Q21 19 Factor 19: can be useful if it fits in with the innovation or the process. So depends on the type of innovation.

Q21 22 Factor 22: See factor 7. Everyone has to do it, so you also have to take an interdisciplinary approach to innovation. Especially if the innovation is intended for multiple disciplines.

Q21 25 Valuing employees: See also factor 32. If they feel they are allowed to innovate and experiment, they will want to keep doing it. If they are punished or taken to task if things are not going fast enough, then a negative mode can develop toward innovation. Instead, you need to reward people for their efforts and for the struggle that comes with innovating.

Q21 30 Factor 13, 14, 30, 34, 36 Learning: also important. Share experiences, evaluate, reflect. Provides incentive to stay motivated in the face of adversity. Innovation is an iterative process: constantly adjust and improve your process (factor 13). This process can be slightly different for each project. It should be servant-driven and therefore not rigid. Keeping an overview (factor 14) is what middle management and the healthcare professional do, and they offer this as input to the board.

Q21 31 Factor 31: Doubtful. Can be important and fine at times, but innovation itself is still done as much as possible in practice itself.

Q21 32 Encourage employees to do it themselves: Innovating, that is, doing things differently is about the people in practice who also have to do it. So you can do or facilitate all kinds of things at the organizational level, but above all you have to encourage people to feel free to just start doing it. That is the basis, because if that is not there, you can think up all kinds of things and want them, but they will not get off the ground. That's where the source of potential success lies. To do this, you have to give them the space to get started on what can help them improve things. That way they also feel that they themselves are in the lead to improve their own work.

Q21 33 Having guts: Both from the board and middle management, but also certainly the employees themselves. And possibly clients/neighbors (factor 15) if they have a role in the innovation. So ties in directly with factor 32: encouraging employees to get started innovating.

Q21 34 Factor 13, 14, 30, 34, 36 Learning: also important. Share experiences, evaluate, reflect. Provides incentive to stay motivated in the face of adversity. Innovation is an iterative process: constantly adjust and improve your process (factor 13). This process can be slightly different for each project. It should be servant-driven and therefore not rigid. Keeping an overview (factor 14) is what middle management and the healthcare professional do, and they offer this as input to the board.

Q21 35 Taking time to learn: You can have innovation as a goal, but if you then put other ambitions first (achieving efficiency requirements, etc.) or if you expect results too quickly from the innovation processes you start, it is not realistic. People must also be given room to make mistakes and take their time. Often innovations are used, for example, to work more efficiently, but that never happens overnight. You have to be able to experiment and try things out.

Q21 36 Factor 13, 14, 30, 34, 36 Learning: also important. Share experiences, evaluate, reflect. Provides incentive to stay motivated in the face of setbacks. Innovation is an iterative process: constantly adjust and improve your process (factor 13). This process can be slightly different for each project. It should be servant-driven and therefore not rigid. Keeping an overview (factor 14) is what middle management and the healthcare professional do, and they offer this as input to the board.

Q22

Q22 1 Very important and that you have to be clear about the innovation ambition, the beckoning perspective

Q22 2

Q22 3 Important

Q22 4 If you have a good idea and mindset then the money will follow naturally, however things also cost money 16.30

Q22 5 It all goes so fast

Q22 6 Then you're already in the anchoring phase, you shouldn't start with this 37.00

Q22 8 Depends on type of innovation

Q22 10 Communicating what's going well and what's not, we (as an organization) often forget to communicate.28.34 and inspire. People never think it's finished enough to communicate. But you can also put the spotlight on small things. You have to communicate from the beginning, because the innovation process is also innovating 29.17 and you have to tell about that

Q22 11 Need to actually get to results you have to get concrete

Q22 12 Toolbox that will all come

Q22 13 Don't get stuck on thinking but definitely start doing

Q22 16 The power of people and family and loved ones

Q22 17 Nice, you generally don't get innovation from knowledge institutions 35.50 nice to share, but most of all do it

Q22 18 We can all do it, but it's about strength 34.50

Q22 19 Learn from each other and inspire each other

Q22 20 Yes especially the vision formation in that,

Q22 22 With this you also create support and bring together all kinds of perspectives: employees, hierarchy, locations, family. Good breeding ground and dialogue at the front before you run 31.30

Q22 23 Not discussed.

Q22 23 Sure provide something, but start doing something first, middle management in elder care those are not the innovators 19.51 more from outside 32.50 frontrunners do it. Middle management you usually have to convince 33.13

Q22 24 I go a bit outside of you, if you are hooked then I will take you right away36.50

Q22 25 commitment

Q22 27 That has to be changed properly administratively. Innovate you have to keep very close as a board. 33.40 Otherwise you are endlessly umming, you want to get off 34.00

Q22 28 That clarity of role for employees that does follow Beyond exploration, not necessarily role, engagement is more important

Q22 32 Link with communication plan, just do it now

Q22 33 Most important guts

Q23

Q23 1 Factor 1: Don't believe in one ambition. You don't have to be a leader in everything, but sometimes be able to follow. Innovating is not a goal an sich. See also factor 27.

Q23 2 That's the framework you need. You need to have a sharp sense of what the innovation should contribute to.

Q23 3 Factor 3: It's okay to describe it, but this can be very broad. It's about the change and whether this falls under innovation, optimization, digitization or automation matters a little less (21:22).

Q23 4 Factor 4: is important, but it doesn't have to be sky-high budgets. However, you need some leeway. You need manpower, but with technical innovations, you also need subscriptions/licenses that you have to take out. Once you've had the start-up costs, look for structural funding. Sometimes it is quite difficult to get a budget, but when you start you often notice that there are also quite a lot of subsidy schemes. So once you get started, there are often opportunities to get additional funding. So finances are often not the problem. The difficulties are more in the manpower to cooperate or in the sustainable funding than in the project funding (23:45). Some (larger) grants are worthwhile, and it is also good that you then commit to something as an organization for a few years. Money can usually be obtained through various schemes.

Q23 5 Factor 5: Flexibility is important when innovating. It is already difficult to set a program for one year, let alone looking 5 years ahead. You can't look that far ahead. Also depends on changes in the world (Covid, funding, etc.). So you have to establish a theme where you want to improve, but you shouldn't pin it down completely.

Q23 6 Is important, especially because people also need to be given time for it and it needs to be clear how innovation is positioned (20:08).

Q23 7 Not discussed further

Q23 8 Factor 8: technical infrastructure you need, but you can also do plenty of innovations that you don't need technical infrastructure with.

Q23 10 You don't necessarily need a communication plan for innovation. You have to see it as part of the regular business (25:37). So rather not a separate communication plan for innovation, but as part of the communication domain. You want it to be part of everybody's regular work.

Q23 11 Factor 11: decision-making is edge conditional. If that's not there, you really don't get anywhere.

Q23 13 Factor 13: provides structure, but you can be flexible in this.

Q23 14 Factor 14: overview you need to follow the project side.

Q23 15 Factor 15 and 25: Important. Finds that when innovating, manpower plays a big role. You need to involve and value people. Family and loved ones also make sure the change climate is there. Regarding involving loved ones and family: when it goes and when the innovation affects them (20:28).

Q23 16 The "soft" side you need. The employees who have to go and do it

Q23 17 Factor 17: it doesn't give the change, but it's nice.

Q23 18 Factor 18: you definitely need to hire all the knowledge, but here there is also the risk in 'because they do it, we do it too' (15:54). Can actually cloud the open mind. This can also be a problem with subsidies or health insurers who want to see certain innovations implemented (16:47). Puts more focus on "the thing" itself, and less on, "what does this really contribute. Did you really have a problem to be solved with this technology? That temptation is very much present.

Q23 19 Factor 19: This is not a "should. You have to have an open mind. These can be external partners, but also internal people with expertise.

Q23 20 Not so explicitly discussed. Believes myself mainly in the action-research methodology: simultaneously looking at what works and what doesn't and fine-tuning during the process. See also factor 34 below.

Q23 23 Factor 23: there is an information 'gap'. That's why you need to provide people with knowledge.

Q23 24 Middle management is crucial to give people the confidence and to bring them into it. Especially middle management has to get innovation 'between the ears'. Innovation belongs to everyone, that idea must live in an organization.

Q23 25 Factor 15 and 25: Important. Finds that people power plays a major role in innovation. You have to involve and value people. Family and loved ones also ensure that the climate for change is created. Regarding involving loved ones and family: when it goes and when the innovation affects them (20:28).

Q23 27 Factor 27: It's important that governance propagates something, but not necessarily that innovation is a priority. You don't have to change for the sake of changing. You have to innovate because it serves a purpose. It is important to work from strategy though (13:20) and this may require innovation/change.

Q23 28 Factor 28: clear roles helps a lot. This clarity comes up a few times. Agreements for middle management and for employees.

Q23 30 Evaluating does, recording is less important. Not necessarily (20:48).

Q23 31 Less important. There are several possibilities. Doesn't believe in that very much.

Q23 32 Factor 32: is important, but you have to give them a framework in this, to make it more likely to succeed. Employees' ideas do need to fit within the vision of the organization, otherwise they have no chance (19:05).

Q23 33 Having guts in important in the process of innovating.

Q23 34 Factor 34: Looking back on mistakes is sometimes more negative. It's better to look forward, because every project is different anyway. There is a risk attached to 'learning from mistakes': we've done this before, it didn't work and we won't do it again. It is also sometimes time-dependent (culture, which people are there) why something succeeds or fails. Sometimes with small change it suddenly succeeds.

Q23 35 Factor 35, 36: Learning is very important. You do want to learn from each other, but mainly looking forward.

Q23 36 Factor 35, 36: Learning is very important. You do want to learn from each other, but especially looking forward.

Q24

Q24 3 not discussed.

Q24 4 There must be money

Q24 10 Says a lot about the importance of informing and communicating but put this factor at not important. Sees it more as an organizational task and director's job

Q24 13 52.40 (personal preference) innovation process is not linear, you can figure it out in advance where do I want to hee, what do I want to achieve. don't put too much time into that, be open to other avenues. 53.00 you always come across bumps in the road

Q24 16 It has to be carried, support is important. Care office tries to integrate this during care procurement by RMP 41.50 is difficult because it deviates from the normal procurement agreements 42.20 Doesn't work well yet. 42.50

Q24 21 Not discussed.

Q24 22 Avoid coming up with everything from the high tower, innovation should actually belong to the whole organization, also doesn't have to mean that everyone has to engage in innovation 38.50 but you have to know that the organization knows that it is important. Shouldn't be an isolated club 40.50 looking from different perspectives 51.00

Q24 24 Middle management should facilitate that and include it in annual reviews .

Q24 27 People only participate if management makes that clear e.g. include in communication to the outside 39.41 e.g. through annual meetings 1 or 2 per year, intranet. Message: be in DNA of our organization, through innovation we can become even better in the future in what we are doing and support older people in independence 40.30. QUOTE

Q24 31 Not discussed.

Q24 32 Being an innovation manager is really a profession. All employees have to be included in innovation, why, what do we want with it, what is needed for that. But to encourage them all to do something with innovation. Leave that with the experts 50.30 You have to have certain competencies and motivation. Everyone has to know why and that not everything works.

Q25

Q25 1 See also factor 27

Q25 2 See also factor 27

Q25 9 Already in so many other cards (factors) there is already the importance of learning and doing something with it, so this factor he felt was already sufficiently covered.

Q25 13 See also factor 16

Q25 16 Hammering on 'innovate from need'. Start a 'customer journey' (?) together with employees and/or clients. Where in your work can some things be improved? Innovation ambitions/themes are then included. Where is it worth innovating? Let the employees/clients themselves prioritize. That way the demand comes from the people themselves. That is an important foundation to build on. It is important that those who will work with the innovation see the importance of it.

Q25 19 Not further discussed

Q25 23 Factor 23 and 24: Middle management has an important role, especially if an innovation needs to be secured. To make sure that all the things you have agreed on are also applied on a day-to-day basis. Middle management is traditionally accustomed to 'looking after the pie', but are not necessarily competent/trained in guiding change.

Q25 24 Factor 23 and 24: Middle management has an important role, especially if an innovation needs to be secured. To ensure that all the things you have agreed on are also applied on a day-to-day basis. Middle management is traditionally accustomed to 'fitting in', but are not necessarily competent/trained in guiding change.

Q25 27 It is crucial that by the board (and middle management) innovation is linked to organizational goals. In what way will innovation contribute to the organizational goals you already had? Then preconditions (time, budget) are also made available because you are contributing to team interests.

Q25 30 Not further discussed

Q25 31 He is critical of all kinds of innovation labs with all kinds of gadgets. From a change point of view, he doesn't really believe in them. He doesn't think people see a gadget and then think, "oh, let's innovate. You shouldn't work from a gadget, but make the match from a need for change in the work process, for example, to deliver the best possible care. You have to think first about the added value you want to deliver, and only then look at a product. You have to put off talking about technology. People also get defensive about this, because it is not about the care and their daily process.

Q25 33 Not further discussed

Q25 35 Factor 35 and 36: Three quarters of a successful change consists of a social innovation, i.e. in the behavioral side. What behavioral change is needed, and what is needed to sustain this behavioral change? Only then are you talking about sustainable impact. That takes a lot of time and space, including learning from mistakes, evaluating and reflecting with each other. What team dynamics are at play? Do you dare to speak things out, make mistakes?

Q25 36 Factor 35 and 36: Three quarters of a successful change consists of a social innovation, that is, in the behavioral side. What behavioral change is needed, and what is needed to sustain this behavioral change? Only then are you talking about sustainable impact. That takes a lot of time and space, including learning from mistakes, evaluating and reflecting with each other. What team dynamics are at play? Do you dare to speak things out, make mistakes?
