# Supplementary material for: Perspectives on managing innovation readiness in long-term care: a Q-methodology study
Source: BMC Geriatr. 2024 Dec 19;24:1017. doi: 10.1186/s12877-024-05572-3 (PMC11658053; doi:10.1186/s12877-024-05572-3)
Supplement: Supplementary file 6 — Additional file 6. [file 12877_2024_5572_MOESM6_ESM.docx]

Q26

Q26 2 You have to make choices, because you can't be doing too much at once. There is not enough staff and budget for that.

Q26 4 Not further discussed

Q26 6 Not further discussed

Q26 12 Not discussed further

Q26 13 See "Perspective..." Here he explains how this is done within his organization, among other things.

Q26 20 About three years ago he was asked more often: are there any new technological gadgets? Innovations very quickly meant healthcare technology. In recent years, technological innovations have therefore been 'pushed' into the department more often from ICT. He then indicated that he would rather see the director come up with a specific question where innovation might be able to help. So innovate from the demand that exists and not from the technology that is available. Then you all have to think about it in the same way. It must originate from the board and management, so that they can coordinate it with the care managers, so that it will also land in that layer of the organization. The vision must also state that innovation is broader than technological gadgets, but can also consist of social or process innovation. He has to keep bringing this to the attention each time. Creating that vision is not that complicated, but the process toward it is valuable. That everyone gets on the same page, that you know where you want to go as an organization and in what way.

Q26 21 It's not necessarily unimportant, but he had to make choices in prioritization. He himself has been working on this issue for years and so have the board and management. They themselves sometimes have to learn to separate healthcare technology (which is often still the focus) from innovation. The ambassadors really come from the care process and they receive an 8-hour training. Among other things, about taking your time, getting people involved, about the different types of innovations there are.

Q26 27 Director is very decisive in propagating the vision and also includes the management in it. Innovation now has continuous attention in his organization and that is very nice. Innovation is now also positioned under the director. An innovator has been appointed to work with new innovations (experimenting, etc.).

Q26 31 He has noticed from his experience that you don't get everyone on board. With the group they have now (the innovator and the ambassadors) they can reach the rest. Have occasionally experimented with a digital market, but that does not yield much. So such a space is not really needed.

Q26 32 Not further discussed

Q27

Q27 1 That's where it starts 26.17 it stands or falls with the ambition that a RMP has 26.20

Q27 4 And then you look very quickly to the director or to the management 26.32 to have the ambition to be engaged in innovation. And then they have to decide to budget for that. That's really the start. Budget needed, what is most essential.

Q27 8 The people have to do it, that you then need even more then will.

Q27 11 Not discussed.

Q27 15 Involving residents.

Q27 16 Getting employees along, getting them excited about it 25.39, also appreciate, reward. People have to do it 27.10 have to be open to it have to be facilitated 27.17. Attitude

Q27 21 not discussed.

Q27 23 not discussed.

Q27 27 And then very quickly you look to the director or to the management 26.32 whether they have the ambition to be engaged in innovation. And then they have to decide to budget for that. That's really the start.

Q27 31 The people have to do it, that you then need even more than that. Not essential

Q27 lector of Health and Welfare of frail elderly at Inholland University of Applied Sciences and Zonnehuisgroep Amstelland.

Q27 Scoping review sent

Q28

Q28 1 Together with should the director indicate why, where do you think, why would you want that and for whom is that good there is the ambition in 25.25

Q28 4 Misunderstanding; organizations think innovation is sequential but there are many more essential parallel processes 0.12. For example that means something for the budget but also for who or what you need in the organization.

Q28 7 Teams by definition have to be interdisciplinary. That's why it is at the back. Setting up teams interdisciplinary is at +1 (22 and 7 belong together)

Q28 10 That's why a communication plan is important, how are you going to engage people. Communication plan is reciprocal, often they are mission plans. Inform about outcomes (not necessarily just results). What have you done with suggestions ed So that people can follow you through open communication.

Q28 18 Sure you do

Q28 21 Organize education not too early, prefer parallel

Q28 27 'Struggles' with middle management and management, it is much more important at all that a director knows his white and black ravens and deploys and invites them to participate. Director should indicate that making mistakes is okay and radiate that.

Q28 28 Invite them to participate

Q28 30 Definitely educate on it, write it up nicely do that in parallel

Q28 33 If you don't have guts, you shouldn't start innovating 23.34 Is a prerequisite, you know you're going to make mistakes and people need to know it's okay. That's what a director has to let people know and feel.

Q28 34

Q28 Sometimes trivial levers, sometimes unintentionally are important, like e.g. sometimes it helps to rearrange a physical space for a short time (it can be decisive because it changes behavior, e.g. different way of meeting, like seduction art). People discover that they will sit differently and then the meeting will go differently. 2.25. Change and innovation are close to each other (the swalum tail) 28.20

Q29

Q29 1 Should actually be the basis, this is where it starts, from which it should be derived 36.46 Don't just do everything but a little bit, like e.g. ZORA, why? Yeah because other organizations are doing it too. You have to start from your own vision.

Q29 3

Q29 8 Just need to be there especially for the technical innovations, however innovation is broad so therefore less important.

Q29 12 Nice to have

Q29 13

Q29 15 Depends on type of innovation, specific solution, sometimes important. Priority is tight labor market, that care can be delivered.44.22 and therefore shift from client to 1 to employee to 1. Actively involve often doesn't come out as much, you have to inform well. Care agreements say client more central....don't necessarily get excited about bedsence employees do

Q29 16 Involve staff to know if the innovation is working or not. What you don't want is staff coming up with something for care. 40.00 Especially in the initial phase, but actually you have to do that all phases.

Q29 19 Innovate with suppliers there is still a lot to gain there. Not just buying products. Actively develop together (like OZO TL) from supplier-customer relationship to co-creation and partnership 41.50

Q29 20 It should not be a trick, you really have to know how you want to do it. Vision on that is still very limited. You have to get a handle on that. If you hire consultants, you still don't know it as an organization. To build capacity e.g. also program management to know how to do that 39.25

Q29 26

Q29 27 And that vision has to be propagated by the director.

Q29 31 Risk of it becoming something separate, it will come, nice to have 42.47 you have to be visible and you don't get that you set up a space. Living lab or house of the future is nice for inspiration or trying things out, not necessary

Q30

Q30 2 It starts with identifying the innovation themes to set priorities

Q30 4

Q30 5 Not necessarily necessary, too managerial, you have to make a culture focused on innovate30.01 better agree on a way of working with each other

Q30 6 Disadvantage can be dichotomy, employees who are working on innovation, and the "poor" souls who have to do the regular work and therefore have to do most of the work. Rather generally ask employees to take position around innovation. It should be part of the regular process so not in addition to the regular organization

Q30 9 Make visible especially the results

Q30 10 Rather than making a communication plan, make the results visible, especially then it becomes inspiring and shows that it is important. With a plan you have even more paper. Rather a few ground rules on how to make a number of things visible.

Q30 12 Who should do that?

Q30 13 Set it up at some point, but start with a climate where people are willing to invent and look for new things

Q30 17 Actively organize internships from MBO and HBO institutions in elder care organizations 1.17.20

Q30 19 Good, but make sure the experience is not invested outside the organization

Q30 24 Above all do it, and live it up

Q30 27 Priority should be getting better, you need innovation for that

Q30 29 Not necessarily necessary, but very meta level

Q30 30 Showing make visible especially the results

Q30 32 Create freedom of thought and action, use the brainpower of employees. Even though they may not be so educated. And appreciate that

Q30 33 Create freedom of thought and action and support that

Q30 34 Open door no brainer
