# Supplementary material for: Perspectives on managing innovation readiness in long-term care: a Q-methodology study
Source: BMC Geriatr. 2024 Dec 19;24:1017. doi: 10.1186/s12877-024-05572-3 (PMC11658053; doi:10.1186/s12877-024-05572-3)

**Additional file 7 :** composite rankings of the statements for perspective 1: supportive role of management


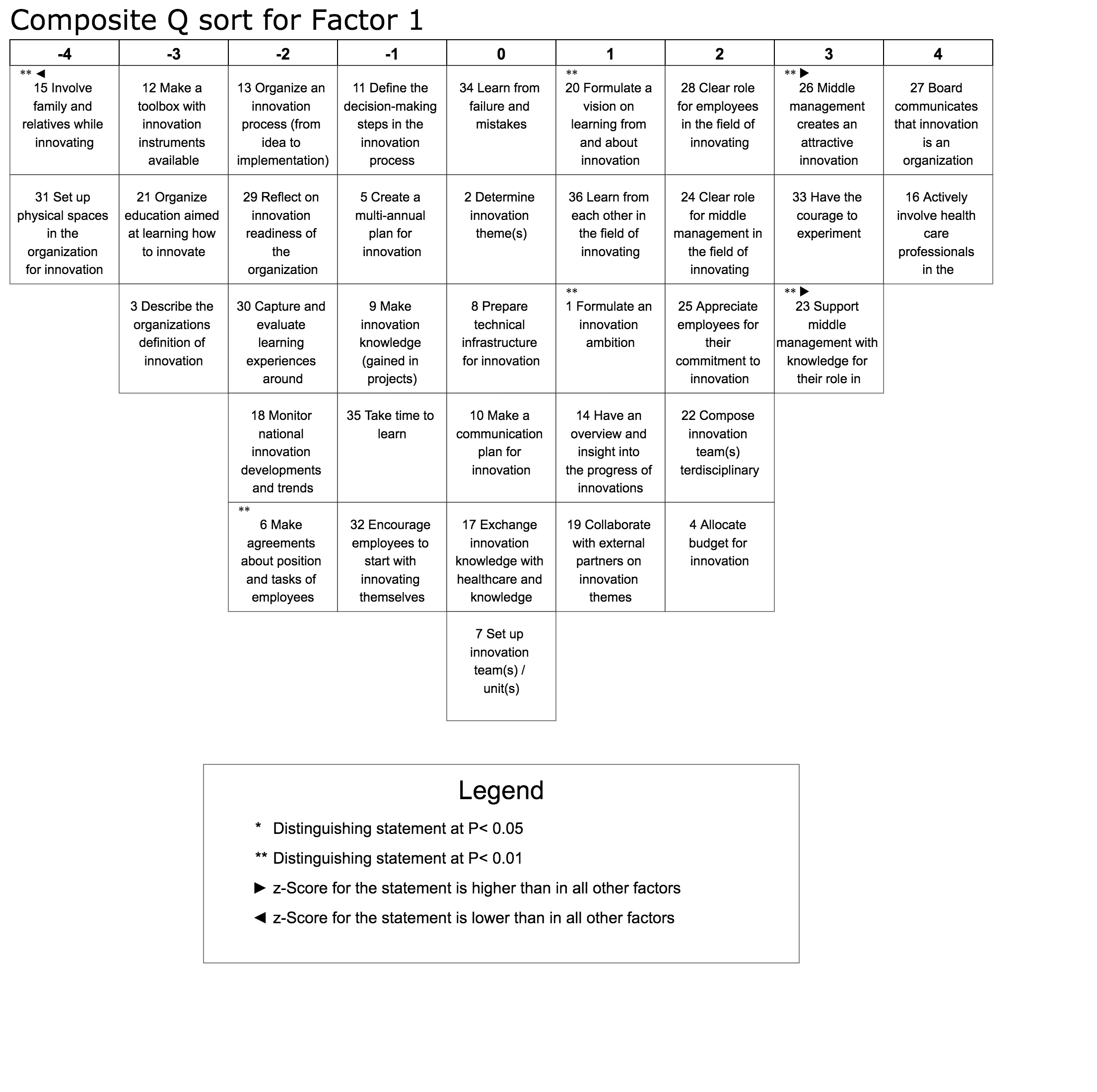


**Additional file 8 :** composite rankings of the statements for perspective 2 : participation of the client (system) and employees


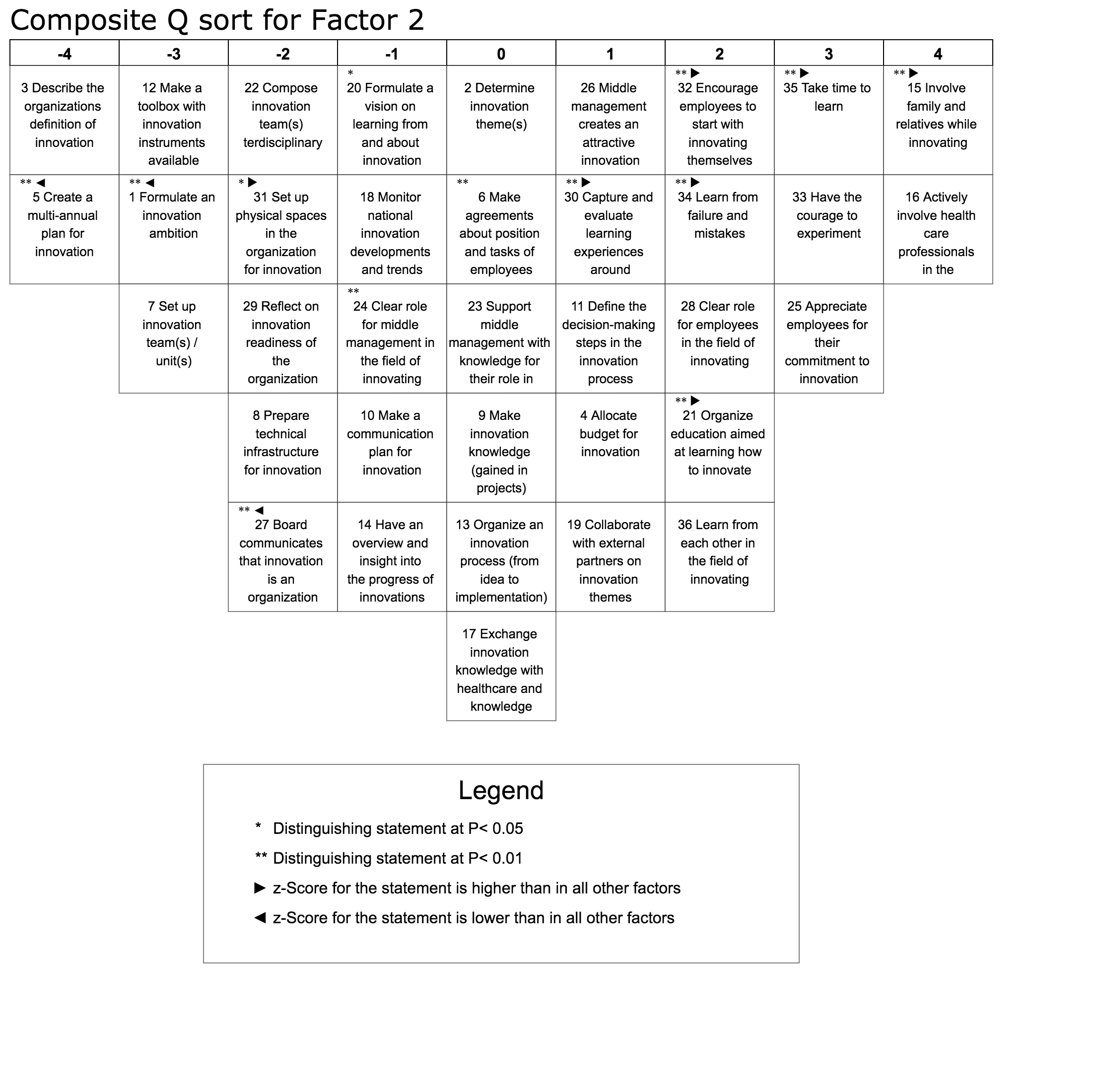


**Additional file 9 :** composite rankings of the statements for perspective 3 : setting the course and creating conditions


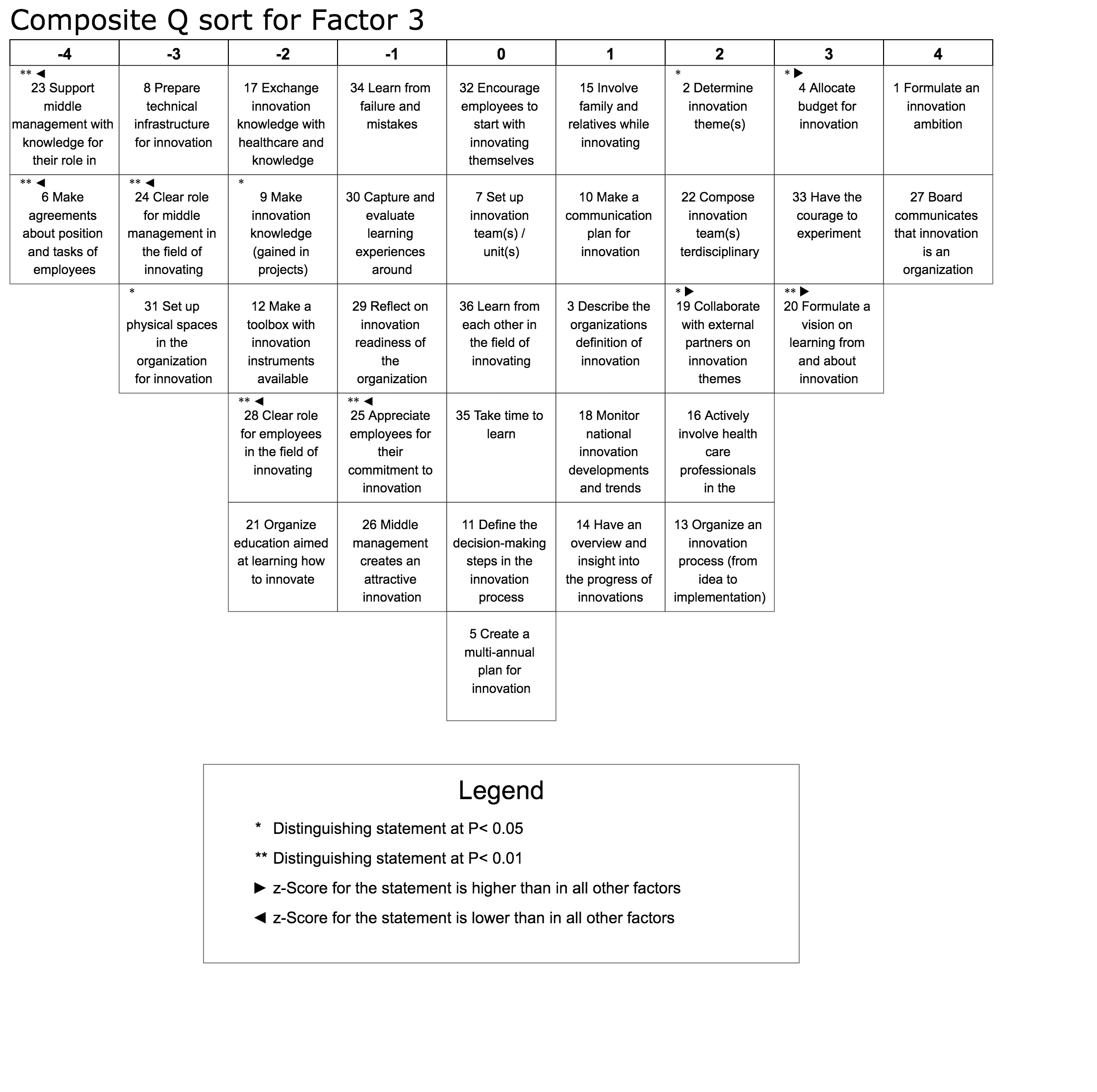


**Additional file 10 :** composite rankings of the statements for perspective 3 : structuring decision-making, roles and responsibilities
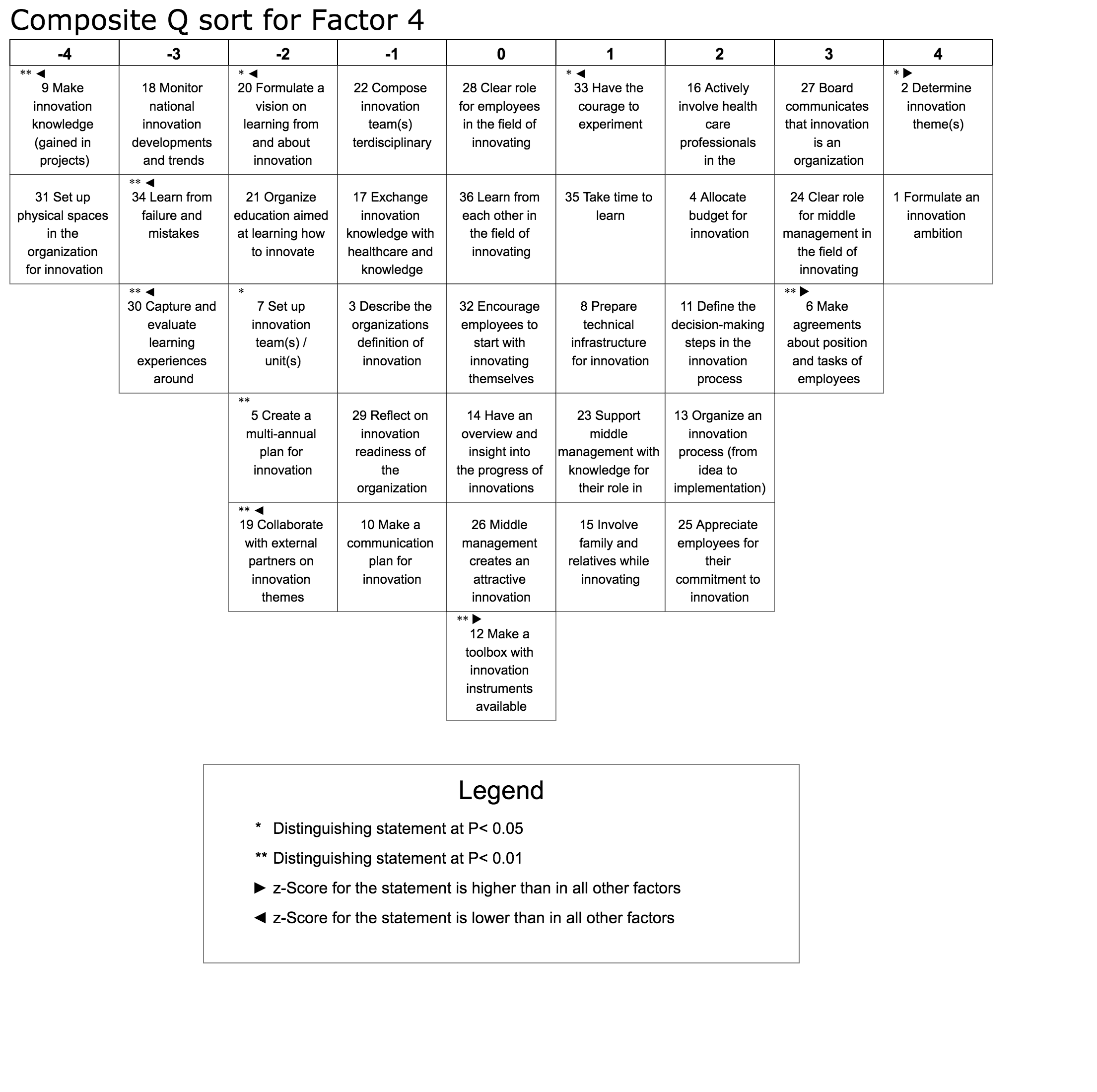

Supplement: Supplementary file 7 — Additional file 7. [file 12877_2024_5572_MOESM7_ESM.docx]
